# Bactericidal Disruption of Magnesium Metallostasis in Mycobacterium tuberculosis Is Counteracted by Mutations in the Metal Ion Transporter CorA

**DOI:** 10.1128/mBio.01405-19

**Published:** 2019-07-09

**Authors:** Landys Lopez Quezada, Sandra Silve, Mark Kelinske, Amir Liba, Constantino Diaz Gonzalez, Martin Kotev, Laurent Goullieux, Stephanie Sans, Christine Roubert, Sophie Lagrange, Eric Bacqué, Cedric Couturier, Alain Pellet, Isabelle Blanc, Marlène Ferron, Fabrice Debu, Kelin Li, Jeffrey Aubé, Julia Roberts, David Little, Yan Ling, Jun Zhang, Ben Gold, Carl Nathan

**Affiliations:** a Department of Microbiology and Immunology, Weill Cornell Medicine, New York, New York, USA; b Evotec Infectious Diseases (Lyon), Marcy l’Etoile, France; c Agilent Technologies, Inc., Wilmington, Delaware, USA; d Evotec Research Informatics (Toulouse), Toulouse, France; e Division of Chemical Biology and Medicinal Chemistry, UNC Eshelman School of Pharmacy, University of North Carolina at Chapel Hill, Chapel Hill, North Carolina, USA; Harvard School of Public Health; University of Alabama at Birmingham; University of Washington; Rutgers University

**Keywords:** CorA, magnesium, mycobacterium, tuberculosis

## Abstract

A defining characteristic of treating tuberculosis is the need for prolonged administration of multiple drugs. This may be due in part to subpopulations of slowly replicating or nonreplicating Mycobacterium tuberculosis bacilli exhibiting phenotypic tolerance to most antibiotics in the standard treatment regimen. Confounding this problem is the increasing incidence of heritable multidrug-resistant M. tuberculosis. A search for new antimycobacterial chemical scaffolds that can kill phenotypically drug-tolerant mycobacteria uncovered tricyclic 4-hydroxyquinolines and a barbituric acid derivative with mycobactericidal activity against both replicating and nonreplicating M. tuberculosis. Both families of compounds depleted M. tuberculosis of intrabacterial magnesium. Complete or partial resistance to both chemotypes arose from mutations in the putative mycobacterial Mg^2+^/Co^2+^ ion channel, CorA. Excess extracellular Mg^2+^, but not other divalent cations, diminished the compounds’ cidality against replicating M. tuberculosis. These findings establish depletion of intrabacterial magnesium as an antimicrobial mechanism of action and show that M. tuberculosis magnesium homeostasis is vulnerable to disruption by structurally diverse, nonchelating, drug-like compounds.

## Abstract (precis)

Antimycobacterial agents might shorten the course of treatment by reducing the number of phenotypically tolerant bacteria if they could kill M. tuberculosis in diverse metabolic states. Here we report two chemically disparate classes of agents that kill M. tuberculosis both when it is replicating and when it is not. Under replicating conditions, the tricyclic 4-hydroxyquinolines and a barbituric acid analogue deplete intrabacterial magnesium as a mechanism of action, and for both compounds, mutations in CorA, a putative Mg^2+^/Co^2+^ transporter, conferred resistance to the compounds when M. tuberculosis was under replicating conditions but not under nonreplicating conditions, illustrating that a given compound can kill M. tuberculosis in different metabolic states by disparate mechanisms. Targeting magnesium metallostasis represents a previously undescribed antimycobacterial mode of action that might cripple M. tuberculosis in a Mg^2+^-deficient intraphagosomal environment of macrophages.

## INTRODUCTION

The global incidence of tuberculosis is trending down, yet drug-sensitive tuberculosis (TB) remains the leading cause of death from infection, and the menace of the disease is rising with the emergence and spread of multidrug-resistant strains (1). Standard treatment of drug-sensitive tuberculosis involves administration of four compounds: isoniazid, rifampin, pyrazinamide, and ethambutol. Prolonged treatment with multiple drugs may be necessary not just to prevent selection for heritable antibiotic resistance but also to overcome phenotypic tolerance (2), which is defined as the ability of some members of a genetically identical bacterial population to survive a drug treatment regimen that quickly kills most of the population under standard conditions. Mycobacterium tuberculosis becomes phenotypically tolerant to most clinically used TB drugs when it enters metabolic states imposed by sublethal stresses that lead to nonreplication (3, 4). With the exception of pyrazinamide, the compounds in the standard drug regimen are most effective on replicating M. tuberculosis and display much less activity when the bacteria slow or cease replication in response to conditions such as hypoxia (5), nutrient starvation (6), and a 4-stress model that combines hypoxia, nutrient limitation, mild acidity, and nitrosative stress (7). Thus, the rise of heritable resistance and the susceptibility of frontline drugs to phenotypic tolerance highlight the need to find new bacterial pathways as targets for treatment of TB. Of particular interest are compounds that kill M. tuberculosis in disparate metabolic states, which may do so by inhibiting more than one target. M. tuberculosis is likely to have lower frequencies of resistance to multitarget drugs than to drugs with single targets of functional relevance.

In recent years, several studies have identified compounds with antimycobacterial activity against both replicating and nonreplicating (NR) cells (8). For example, nitazoxanide, an FDA-approved drug for other infections, kills both replicating and nonreplicating M. tuberculosis *in vitro* (9, 10), as does the metal-chelating agent 8-hydroxyquinoline (8HQ) (11, 12). TCA1 targets the decaprenyl-phosphoryl-β-d-ribofuranose oxidoreductase DprE1, which can be mycobactericidal under replicating conditions, and MoeW, an enzyme involved in molybdenum cofactor biosynthesis, which may account for the compound’s mycobactericidal action against nutrient-deprived, nonreplicating M. tuberculosis (13). SQ109 disrupts synthesis of menaquinone and can induce the collapse of the transmembrane proton gradient, killing replicating M. tuberculosis H37Rv cells and nonreplicating streptomycin-starved M. tuberculosis 18b cells (14). We have recently described cephalosporins that kill M. tuberculosis *in vitro* both when it is replicating and when it is not (15). However, at present, there are a limited number of drugs that are FDA approved for the treatment of tuberculosis and that are reported to kill both replicating and nonreplicating M. tuberculosis *in vitro*. These include rifampin, bedaquiline, and fluoroquinolones, but their activity against nonreplicating M. tuberculosis *in vitro* can be an artifact of compound carryover from nonreplicating to replicating conditions in such assays (7).

Here we report 3 new 4-hydroxyquinolines (4HQs) and a barbituric acid (BA) derivative with potent antimycobacterial activity against both replicating and nonreplicating M. tuberculosis whose activity against nonreplicating M. tuberculosis is not attributable to compound carryover. The compounds appear to have multiple molecular targets, because they kill replicating and nonreplicating M. tuberculosis by different mechanisms. While we have not identified the targets, we have uncovered disruption of magnesium metallostasis as a novel mechanism of action of the compounds against replicating M. tuberculosis.

## RESULTS

### Identification of structurally diverse compounds with bactericidal activity against replicating and nonreplicating M. tuberculosis.

**FIG 1:**
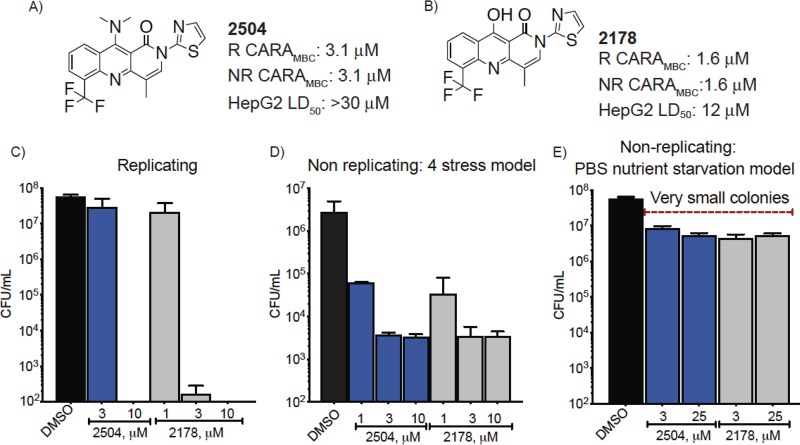
Structure and biological activity of antimycobacterial agents. (A and B) The activity of (A) 2504 and (B) 2178 against M. tuberculosis cells that were replicating (R) or nonreplicating (NR) in the 4-stress model as assessed by charcoal-agar resazurin assay (CARA) and their activity against human HepG2 cells. The CARA_MBC_ is the concentration of compound used to reduce the signal to <1% of the fluorescent signal of the DMSO controls. (C to E) CFU/ml after 7 days of compound exposure of M. tuberculosis under (C) replicating conditions, (D) NR conditions in the 4-stress model, and (E) NR conditions in the nutrient starvation model. Data are means ± SD from one experiment representative of at least 3 independent experiments performed in triplicate using DMSO as the vehicle control.

Two subsets of the Sanofi compound collection, one containing 674,000 compounds and the other 90,000 compounds, were used in two independent screens against either a dual auxotrophic strain of M. tuberculosis (16) or Mycobacterium smegmatis. These screens discovered two different chemical scaffolds with antimycobacterial activity, both of which were active against virulent M. tuberculosis H37Rv: tricyclic 4-hydroxyquinolines (4HQs) (Fig. 1) and a barbituric acid (BA) derivative (see Fig. S1A in the supplemental material). Most of our studies focused on two 4HQs, termed 2504 (Fig. 1A) and 2178 (Fig. 1B). Charcoal-agar resazurin assays (CARAs) were conducted to provide an estimation of cidality and ensure that the activity against nonreplicating cells was not attributable to compound carryover from the nonreplicating stage of the assay to the outgrowth stage (7). CARAs demonstrated that after a 7-day exposure to 2178 at 1.6 μM or to 2504 at 3.1 μM, the compounds killed at least 2 log_10_ of replicating M. tuberculosis and that the same concentrations had the same impact on M. tuberculosis rendered nonreplicating (NR) by a 4-stress model that combines hypoxia, nutrient limitation, mild acidity, and a source of reactive nitrogen intermediates (17, 18) (Fig. 1A and B).

10.1128/mBio.01405-19.2 FIG S1 Structure, antimycobacterial activity, and cytotoxicity of SAR1 and 4HQ 2150. (A) SAR1 and (B) 2150 were tested against M. tuberculosis under replicating (R) or nonreplicating (NR) conditions in the 4-stress model and against human HepG2 cells. The activity of SAR1 against replicating M. bovis BCG was determined by IC_80_. Data are means from one of two similar experiments, each in triplicate. Download FIG S1, PDF file, 0.4 MB. Copyright © 2019 Lopez Quezada et al. 2019 Lopez Quezada et al. This content is distributed under the terms of the Creative Commons Attribution 4.0 International license.

Guided by the semiquantitative CARA results above, we went on to quantify the reduction in CFU. Under replicating conditions at 10 μM, 2178 and 2504 reduced the CFU by >5 log_10_ to below the limit of detection within 7 days (Fig. 1C). In the 4-stress nonreplicating model at 10 μM, both compounds reduced the CFU by 3 log_10_ within 7 days (Fig. 1D). In the nutrient-starved model of nonreplication, 2504 and 2178 reduced the CFU by 1 log_10_ (Fig. 1E) following a 7-day exposure to the compounds. A third tricyclic 4HQ, 2150, was also active against both replicating and nonreplicating M. tuberculosis (Fig. S1B), but because of its poorer solubility, its use was limited to selection of resistant mutants. The BA derivative SAR1 had a CARA minimal bactericidal concentration (CARA_MBC_) of 0.78 μM against both replicating and nonreplicating M. tuberculosis (Fig. S1A).

### Resistant clones share mutations in *corA*.

The novelty of the structures among reported mycobactericidal compounds encouraged us to explore their mechanism of action. Unlike 8HQs, 4HQs lack a hydroxyl at the C-8 position to coordinate with the ring nitrogen and chelate metals (19). Therefore, we anticipated that the tricyclic 4HQs had a different mode of activity. While other BA derivatives similar to SAR1 have been reported to display antibacterial activities (20), to our knowledge, there are no conclusive reports of their mode of action against M. tuberculosis or other bacteria. Accordingly, we proceeded to select mutants resistant to these compounds under replicating conditions.

**FIG 2:**
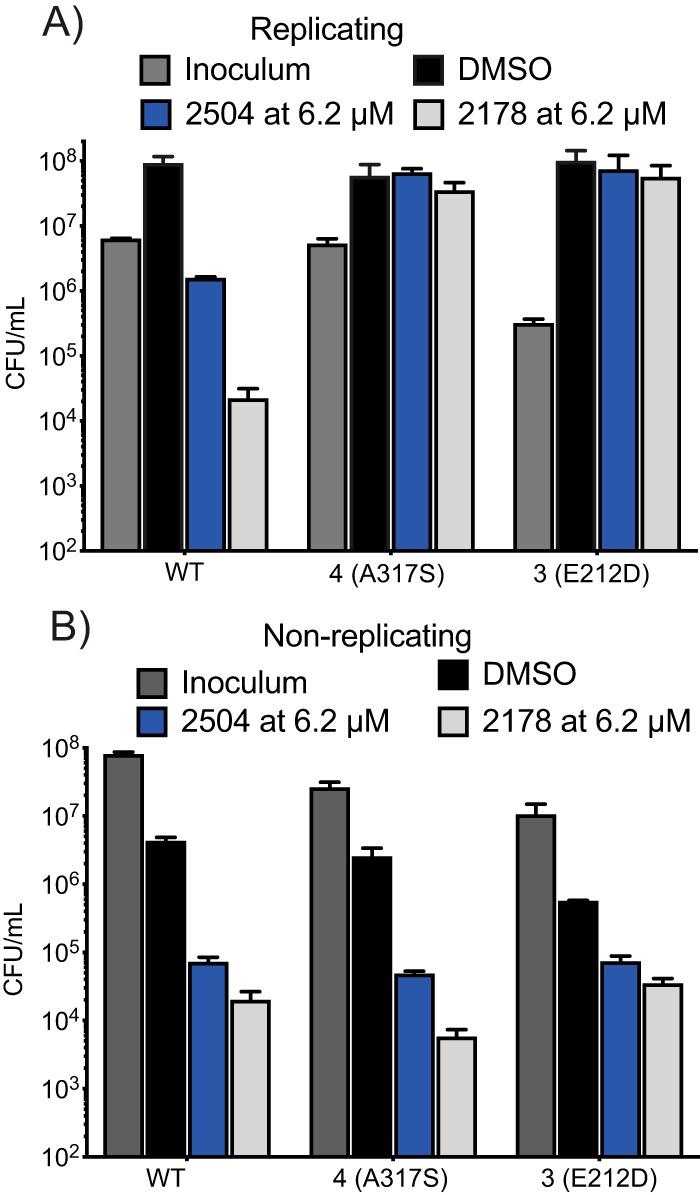
Resistance of CorA mutants to cidality of 2504 and 2178 under replicating (R) conditions but not NR conditions. WT or mutant strains of M. tuberculosis were exposed to compounds at 6.2 μM for 7 days under (A) replicating and (B) nonreplicating conditions in the 4-stress NR model, and the remaining CFU/ml were determined. Data are means ± SD from one of two similar experiments, each in triplicate.

All four compounds were characterized by a low frequency of resistance in M. tuberculosis (4HQs) or Mycobacterium bovis BCG (SAR1), ranging from 1.1 × 10^−8^ to 5.6 × 10^−9^ for the 4HQs and 4 × 10^−8^ for SAR1 (Table 1). After repeated efforts involving incubations of up to 2 months on bacteriologic agar plates containing 4HQs at concentrations ranging from 2 to 8× their broth MIC, we could only isolate 7 M. tuberculosis clones partially resistant to 4HQs. Additionally, four SAR1-resistant BCG clones displayed a consistent MIC of >2 μM, corresponding to >10× the broth MIC of the compound (Table 1). Whole-genome sequencing of the 7 M. tuberculosis clones and 2 of the BCG clones revealed single nucleotide polymorphisms (SNPs) in *rv1239c*, a gene encoding the putative Mg^2+^/Co^2+^ cation transporter, CorA. Amplification and sequencing of *corA* in the two remaining BCG clones revealed SNPs in *corA* as well. No other mutations were found in 5 of the 7 4HQ-resistant M. tuberculosis clones. The additional mutations found in some M. tuberculosis clones did not appear to impact the sensitivity to the compounds relative to the clones whose only mutation was in *corA*. The BCG SAR1-resistant clones displayed no change in susceptibility to isoniazid, bedaquiline (TMC207), or cyclohexyl-griselimycin (21) (see Table S1 in the supplemental material). Similarly, although the 4HQ-resistant mutants showed a 4- to 8-fold increase in MIC (Table 2), the resistance was specific to the 4HQs as they had wild-type susceptibility to rifampin and moxifloxacin (Table 2). Clone 6(D285G) consistently grew more slowly than the WT strain and was 2- to 4-fold more sensitive to rifampin. The seemingly modest 4-fold increase in MIC of 4HQs corresponded to complete protection from the cidal effect of a 7-day exposure to 2178 or 2504 at 6.2 μM (Fig. 2A). In contrast, the CorA mutations did not protect M. tuberculosis from the cidal effect of either of these 4HQs under nonreplicating conditions (Fig. 2B). These findings suggested that (i) the mechanisms of action of 4HQs against replicating and nonreplicating M. tuberculosis are different, and (ii) the mechanism of action of 4HQs and SAR1 against replicating M. tuberculosis and replicating BCG, respectively, may be related to disruption of metal homeostasis.

**TABLE 1:** Selection of resistant mycobacterial mutants

Clone mutational analysis result with the indicated compound, species (μM concn), FOR^*a*^											
2504, *M. tuberculosis* H37Rv (12.4), 6.8 × 10^–8^			2178, *M. tuberculosis* H37Rv (8), 5.6 × 10^–9^			2150, *M. tuberculosis* H37Rv (10), 1.1 × 10^–8^			SAR1, BCG (2 and 8), 4 × 10^–8^		
Clone	CorA mutation	Other gene(s)^*d*^	Clone	CorA mutation	Other gene	Clone	CorA mutation	Other gene	Clone	CorA mutation	Other gene(s)
1	E212D	None	4	A317S	*rv1922* Y153^*b*^	5	L229V	None	1	A317S	*vapB48*
2	D285N	*rv1520* S317^+^ *ppsE* W1428R *rocE* V270^+^				6	D285G	None	2	A317S	*corA* only^*c*^
3	E212D	None				7	L229V	None	3	L229V	*fadD30*, *ppsB/C* deleted
									4	G299S	*corA* only^*c*^

a FOR, frequency of resistance.

b Stop codon.

c For this clone, *corA* alone was sequenced; whole-genome sequencing was not carried out.

d “+” denotes a frameshift mutation.

**TABLE 2:** MICs of resistant *M. tuberculosis* clones grown under replicating conditions^*a*^

Selection compound	Clone	aa change of *corA* SNP	MIC (μM) of:			
			2504	2178	Rif	Moxi
2504	1	E212D	12.5	6.25	0.16	NT
	2	D285N	12.5	NT	0.08	0.08
	3	E212D	12.5	12.5	0.08	0.08
2178	4	A317S	12.5	6.25	0.08	0.16
WT	H37Rv		3.1	1.6	0.08	0.16

a aa, amino acid; Rif, rifampin; NT, not tested; Moxi, moxifloxacin.

10.1128/mBio.01405-19.9 TABLE S1 Selective resistance to SAR1 by BCG *corA* mutants. Download Table S1, DOCX file, 0.1 MB. Copyright © 2019 Lopez Quezada et al. 2019 Lopez Quezada et al. This content is distributed under the terms of the Creative Commons Attribution 4.0 International license.

The region encoding CorA in M. tuberculosis and M. bovis is monocistronic (see Fig. S2A in the supplemental material), so the *corA* mutations were likely to affect CorA itself rather than neighboring genes. However, *rv1239c* is reportedly nonessential to replicating cells (22). Thus, it seemed unlikely that CorA is the target of 4HQs or SAR1. Instead, we considered that altered function of CorA could diminish the cidal impact of the 4HQs and SAR1. We reasoned that the compounds’ mechanism of action might come into focus if we could predict the functional consequences of the resistance-conferring mutations in CorA.

10.1128/mBio.01405-19.3 FIG S2 Homology modeling of the mycobacterial CorAs with the CorA from T. maritima. (A) Position of *corA* in the M. bovis (Mbv) and M. tuberculosis (Mtb) genomes. (B) Primary amino acid sequence alignment of T. maritima (Tmar), M. tuberculosis, M. bovis, and M. smegmatis (Msmeg) CorA. Mutations in resistant clones are in blue. Green triangles mark metal binding site M1; burgundy triangles mark cytoplasmic metal binding site M2. (C) MD analysis of the Ser317 side-chain H-bonds in the five CorA monomers. Orientation of the Ser317 side chains in the CorA channel extracted from a 100-ns MD trajectory snapshot revealed H-bonds with an i-4 Ala313 backbone carbonyl oxygen. (D) The same frame as in panel C viewed from the intracellular part of the protein, Ala313 carbonyl oxygen atoms are depicted as red spheres. (E) Frequency of the hydrogen bond formation between the hydroxyl of S317 and the carbonyl oxygen atom (S317–O–**H^…^O **=** **C–) at positions i-4 (Ala313 [in red dots]) and i-3 (Gly314 [in green dots]) during 200-ns MD simulation. Distances vary between 1.7 and 2.1 Å, approximately. (F) Mapping of the L229 location on the model of M. tuberculosis CorA using the closed conformation of the TmCorA structure in PDB 4i0u. Download FIG S2, PDF file, 0.2 MB. Copyright © 2019 Lopez Quezada et al. 2019 Lopez Quezada et al. This content is distributed under the terms of the Creative Commons Attribution 4.0 International license.

### Predicted impact of mutations in the pentameric CorA protein.

**FIG 3:**
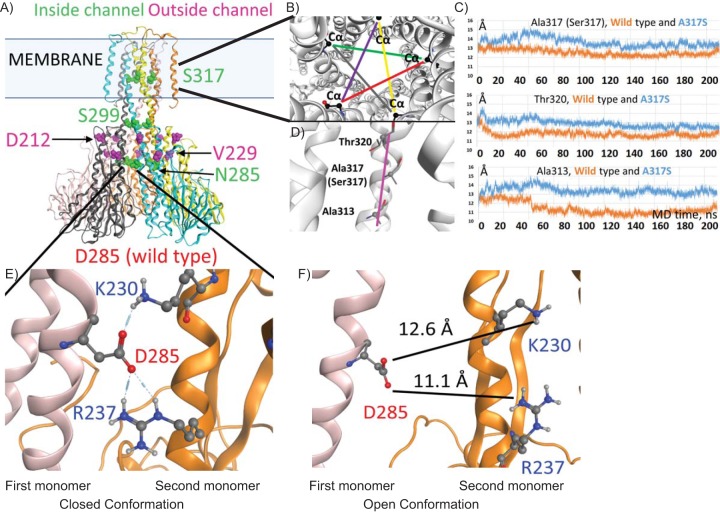
Potential mechanism of CorA-mediated resistance to 4HQs. (A) Model of M. tuberculosis CorA based on T. maritima crystal structures and Molecular Operating Environment (MOE) homology program prediction of the structural location of amino acid residues affected by *corA* mutations in the M. tuberculosis-resistant clones. (B) Changes in the channel diameter around the mutated residue. Five Cα distances that are related to the channel diameter are shown in different colors (as viewed from the periplasmic side, top-down). (C) Plots showing the average value for the five distances (in panel B) taken at each 20-ps MD trajectory frame. (D) The part of the helix facing the channel (magenta) shows A317S and neighboring residues Thr320 and Ala313. (E) The predicted H-bonding between D285 and the adjoining monomer in the closed conformation. (F) Model of the D285 mutation of M. tuberculosis CorA using the open conformation PDB 3jcg from the TmCorA structure, predicting an increase in distance between D285 and the neighboring monomer.

Using the crystal structure of CorA from Thermotoga maritima (23) (PDB 4i0u) as a model, we queried the potential functional significance of the resistance-conferring mutations in mycobacterial CorA. M. tuberculosis CorA has 99% sequence identity with M. bovis CorA (Mb1271c), 63% with M. smegmatis CorA (MSMEG_5056), and 26% with T. maritima CorA (Fig. S2B). All four sequences share the cytoplasmic metal binding sites (Fig. S2B, triangles) and the GMNF metal recognition sequence between transmembrane (TM) helices 1 and 2. The M. tuberculosis and BCG CorA mutations that confer resistance to 4HQs or SAR1 are marked in Fig. S2B (blue squares). Mutated residues S317, S299, and N285 are predicted to line the inside of the channel in its closed conformation, while mutated residues D212 and V229 are modeled as residing in the outer acidic ring (Fig. 3A).

Molecular dynamics (MD) simulations of the M. tuberculosis CorA molecule in a dimyristoyl phosphatidylcholine (DMPC) bilayer were performed in order to predict the consequences of the A317S mutation in the TMD of the CorA homopentamer. MD analysis of the hydrogen bonding of the mutated S317 side chain (Fig. S2C) showed preferred H-bond formation to the backbone carbonyl of the same helix at index (i-) residue position i-3 or i-4 (Fig. S2D and E). Ballesteros et al. studied Protein Data Bank (PDB) structures containing α-helices, and for serines present in the middle of helices, they identified a high propensity for a similar hydrogen bonding pattern to that predicted here between serine side chains and backbone oxygen atoms of the same helix (24). The authors found that these additional hydrogen bonds altered the torsion angles of the helix (24), and so we performed further analysis to predict the impact of the A317S mutation on the diameter of the CorA transmembrane channel (see Movie S1 in the supplemental material). The average distances between the 5 Cαs of S317 (Fig. 3B), for the mutated CorA, were predicted to be higher than the corresponding distances between the Cαs of A317 in the wild-type protein (Fig. 3C). We also considered A313 and T320, which reside, respectively, just before and just after the A317 (or S317) position on the same face of the helix (Fig. 3D). The average distances between the 5 Cαs of A313 and T320 were also predicted to be higher in the mutated CorA protein than in the wild-type homopentamer (Fig. 3C). On average, the distances were higher by 1 to 2 Å.

10.1128/mBio.01405-19.10 MOVIE S1 Supplemental movie. Download Movie S1, MOV file, 2.9 MB. Copyright © 2019 Lopez Quezada et al. 2019 Lopez Quezada et al. This content is distributed under the terms of the Creative Commons Attribution 4.0 International license.

Comparison of the potential impact of the N285D mutation on the channel in its closed conformation (Fig. 3E, lower panel) and in its open conformation (Fig. 3F) led us to predict that the mutation would have its greatest effect on the interactions of N285 with K230 and R237 in the neighboring helix when the channel is closed, considering that the opening of the channel spreads these helices too far apart for hydrogen bonding to take place (Fig. 3F). Similarly, the L229V mutation on the outside of the channel is likely to be consequential when the channel is closed, because closure allows L229 to interact with hydrophobic residues in the adjoining helix (Fig. S2F).

Overall, the modeling predicted that the resistance-conferring mutations destabilize the closed conformation of CorA. Given that CorA’s predicted function is to allow regulated metal influx (25), this inference led us to hypothesize that the mutations could favor increased metal influx, which in turn might mitigate the effect of deleterious loss of available metal(s) resulting from the action of the compounds.

### CorA as a mediator of resistance.

To test the hypothesis that resistance to 4HQs arose through augmented metal influx through mutant CorA, we used the CorA inhibitor hexaammine cobalt (HexaCo), a hydrated magnesium analog (26) (Table 3). HexaCo binds the outer portion of the transmembrane domain of T. maritima CorA (TmCorA) (27) and blocks passage of cations through the channel. On its own, HexaCo had no impact on the viability of WT M. tuberculosis, nor did it affect the activity of the 4HQ 2504 against WT M. tuberculosis (Table 3). In contrast, HexaCo restored the sensitivity of the M. tuberculosis 3(E212D) and 6(D285G) CorA mutants to 2504 and 2178 without affecting the M. tuberculosis strain’s sensitivity to rifampin (Table 3).

**TABLE 3:** Sensitivity of CorA mutants in the presence of HexaCo
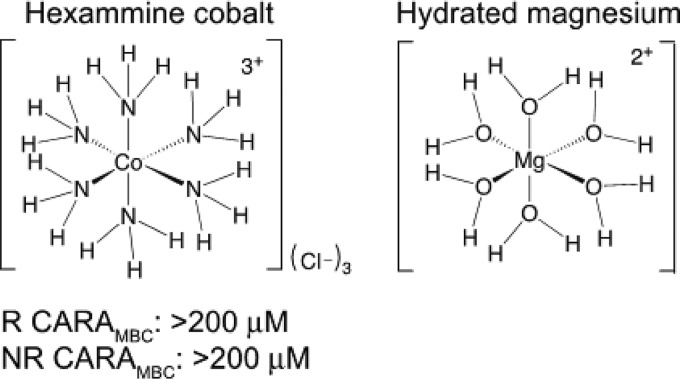

*M. tuberculosis* strain with (+) and without (–) 100 μM HexaCo	CARA MBC (μM) of:			
	Rif	2504	2178	HexaCo^*a*^
WT–	0.31	3.1	3.1	>1,000
WT+	0.31	3.1	3.1	NA
3(E212D)–	0.31	12.5	12.5	>1,000
3(E212D)+	0.31	3.1	3.1	NA
6(D285G)–	0.16	6.2	12.5	>1,000
6(D285G)+	0.16	3.1	3.1	NA

a NA, not available.

The lack of a growth-inhibitory or cidal effect of the CorA blocker HexaCo on WT M. tuberculosis supported the supposition that CorA was not the target of 4HQs or SAR1. To further explore this hypothesis, we tested the impact of transposon-mediated disruption of *corA*. For comparative purposes, we also tested transposon mutants of *mgtE* and *mgtC*, which encode other classes of metal transporters (see Fig. S3A in the supplemental material). All three transposon mutants were viable and displayed no growth phenotype, confirming the reported nonessentiality of *corA*, *mgtE*, and *mgtC* based on survival of transposon mutants in pooled cultures (22). Disruption of the *corA* and *mgtE* genes had a marginal effect on the cidality of 2178, 2504, and SAR1 but not rifampin in the semiquantitative CARA (Fig. S3B). However, quantitative CFU assays indicated that loss of CorA resulted in more killing of M. tuberculosis by 2504 and SAR1 than was seen with WT M. tuberculosis. There was no increased sensitivity to rifampin (Fig. S3B). Conversely, overexpression of c-Myc fusion proteins of CorA bearing the A317S or L229V mutations, but not the wild-type CorA allele, in BCG led to a >10-fold increase in the 50% inhibitory concentration (IC_50_) of SAR1 (Fig. S3C), even though the mutant and WT CorA proteins were expressed at comparable levels (Fig. S3D). Overexpression of MgtE in BCG increased the resistance to SAR1. Taken together, these results suggested that WT CorA at WT levels partially protects mycobacteria from the 4HQs and SAR1, while mutant CorA at WT or higher levels protects mycobacteria from these compounds to a much greater extent.

10.1128/mBio.01405-19.4 FIG S3 Sensitivity of transgenic M. tuberculosis or BCG to compounds. (A) Transposon insertion sites (triangle) and the number of nucleotides from the start site where the insertion cassette begins. (B) Sensitivity of transposon mutants to the indicated compounds. Shown is the impact of *corA* disruption on CFU/ml in response to 7 days of exposure to 2504, SAR1, and rifampin. Data are means ± SD from one of two similar experiments, each in triplicate. M. tuberculosis H37Rv *corA*::*tn*, *mgtE*::*tn*, and *mgtC*::*tn* mutants, a generous gift from Deborah Hung (A. K. Barczak, R. Avraham, S. Singh, S. S. Luo, et. al., PLoS Pathog 13:e1006363, 2017, https://doi.org/10.1371/journal.ppat.1006363), were supplemented with 25 μg/ml kanamycin. (C) IC_50_ of SAR1 in BCG expressing c-MYC-tagged WT CorA or the mutant form of CorA. The known ion channel MgtE was also overexpressed, and DprE served as a negative control. (D) Western blot indicating levels of c-MYC–CorA fusion protein in BCG ectopically expressing different CorAs. To express these proteins in BCG, the salicylate-inducible promoter (gene *rv0560c*) was PCR amplified and inserted between the NotI and EcoRI sites of pSC859, a shuttle vector containing the origins of replication for E. coli and M. tuberculosis and kanamycin resistance as a selection marker, kindly provided by Evelyne Liauzun. The fusions c-MYC–CorA, c-MYC–MgtE, and c-MYC–DprEI were PCR amplified and cloned under the control of the salicylate promoter between the NdeI and HindIII sites. Download FIG S3, PDF file, 0.9 MB. Copyright © 2019 Lopez Quezada et al. 2019 Lopez Quezada et al. This content is distributed under the terms of the Creative Commons Attribution 4.0 International license.

In short, CorA does not appear to be the target of the compounds, but instead mitigates the compounds’ cidal effect against replicating M. tuberculosis. This conclusion encouraged us to measure the metal content of WT and CorA mutant M. tuberculosis with and without exposure to 4HQs.

### 4HQs disrupt M. tuberculosis metallostasis.

To measure the impact of the compounds and the CorA mutations on M. tuberculosis, we took two complementary approaches. Inductively coupled plasma mass spectrometry (ICP-MS) detects total metal contents of samples with high sensitivity and specificity. There are very few reported measurements of total levels of a given metal in mycobacteria (28) and none to our knowledge in nonreplicating M. tuberculosis. Preparation of the sample destroys macromolecules, releasing metals bound to them. In contrast, in the second approach, the activity of exogenous, Mg^2+^-dependent glycerol kinase allowed the specific estimation of the level of free Mg^2+^ in the mycobacterial lysates.

**FIG 4:**
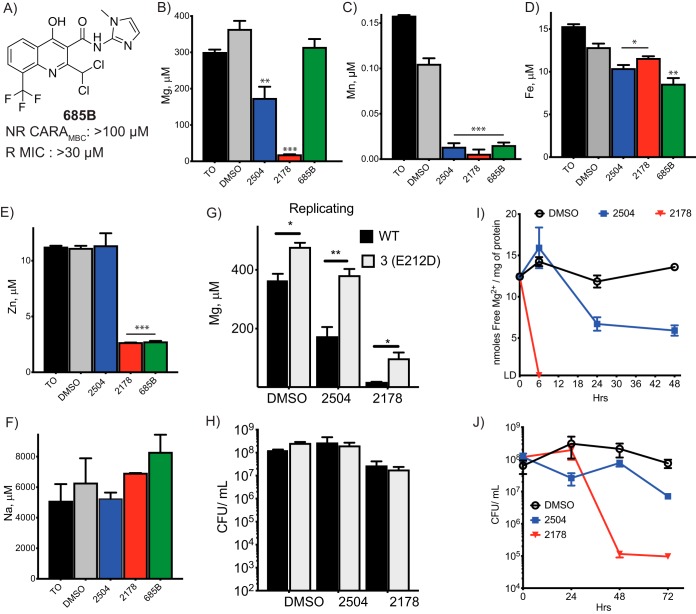
Disruption of metallostasis in cells exposed to 4HQs. Approximately 10^9^ replicating M. tuberculosis cells from WT and resistant strains were exposed to compounds at 25 μM for 24 h, including to the inactive 4HQ (A). Cells were harvested, and the total metal content was analyzed by ICP-MS for (B) magnesium, (C) manganese, (D) iron, (E) zinc, and (F) sodium. The method detects metals in both free and bound forms. Values are expressed as concentrations in the 30% nitric acid extract in which ppm (μg/ml) or ppb (ng/ml) were converted to μM. Comparison of (G) magnesium levels and (H) CFU/ml of WT and *corA* mutant 3(E212D). (I) Approximately 4 × 10^8^ CFU/ml of M. tuberculosis were exposed to the indicated compounds at 25 μM for 6, 24, 48, or 72 h. Cells were harvested to measure labile intracellular levels of free Mg^2+^ by glycerol kinase assay and (J) the change in CFU/ml over time. Tests of statistical significance are indicated for comparisons between (B to F) WT M. tuberculosis treated with DMSO or the indicated compounds or (G) between WT and 3(E212D) M. tuberculosis in response to vehicle or compound exposure. *, *P*< 0.02; **, *P* < 0.002; ***, *P* < 0.0002. Data are means ± SD from one of two similar experiments, each in triplicate.

We incubated M. tuberculosis with dimethyl sulfoxide (DMSO) vehicle control, the mycobactericidal 4HQ 2504 or 2178, or an inactive 4HQ, 685B (Fig. 4A), for 24 h (replicating M. tuberculosis) or for both 24 and 48 h (nonreplicating M. tuberculosis). ICP-MS analysis revealed that incubation of replicating M. tuberculosis with 2504 halved the total magnesium content in the samples, while 2178 reduced the magnesium content almost to background levels (Fig. 4B). In contrast, 685B had no significant effect on magnesium levels. Taking into account the CFU used to generate the samples, the approximate total number of moles of metal in the samples, and an average volume of an M. tuberculosis cell of 8.3 μm^3^ (29), the total magnesium content of a DMSO-treated M. tuberculosis cell after 24 h in fresh 7H9 medium would be estimated to be ∼60 mM, although as described below, most of it appears to be bound. In comparison, total Mg^2+^ content of the majority of mammalian cells ranges from 17 to 20 mM (30), and Escherichia coli cells contain approximately 30 mM total magnesium (31). Applying the same calculations to compound-treated cells, magnesium levels fell to about 13 mM in an M. tuberculosis cell treated with 2504. These estimates assume that the compounds do not affect cell size.

All three 4HQs, including the nonmycobactericidal compound 685B, reduced total cellular manganese by 10-fold compared to the DMSO-treated samples (Fig. 4C). Iron levels were reduced by 10 to 20% below the levels in DMSO-treated cells by the active 4HQs and reduced by 35% upon exposure to the nonmycobactericidal 4HQ 685B (Fig. 4D). 2178 and 685B reduced zinc levels to a third of those in samples from M. tuberculosis cells exposed to DMSO alone (Fig. 4E), while zinc levels were unaffected by exposure to 2504 (Fig. 4E). Levels of sodium were unaffected by any of the compounds (Fig. 4F).

In samples from nonreplicating M. tuberculosis, total levels of magnesium (see Fig. S4A in the supplemental material) and manganese (Fig. S4B) decreased by ∼50% in response to 48-h treatment with 2178 or 2504. Iron levels in samples from nonreplicating cells were not significantly affected by 2178 compared to the samples from DMSO-treated cells at the corresponding time point but rose in the presence of 2504 (Fig. S4C).

10.1128/mBio.01405-19.5 FIG S4 ICP-MS analysis of metals in nonreplicating cell exposed to 4HQs. Using the 4-stress model, nonreplicating M. tuberculosis cells were exposed to 2504 or 2178 for 24 and 48 h, and the total contents of (A) magnesium, (B) manganese, and (C) iron were measured. Comparisons were made between the DMSO controls and the compound-treated cells at the corresponding time points. *, *P* < 0.002; **, *P* < 0.0002; ***, *P* < 0.00002. Data are means ± SD of triplicates. Forty-eight-hour experiments are representative of two similar experiments, while the 24-h experiment was conducted once. Download FIG S4, PDF file, 0.1 MB. Copyright © 2019 Lopez Quezada et al. 2019 Lopez Quezada et al. This content is distributed under the terms of the Creative Commons Attribution 4.0 International license.

In sum, 4HQs variably affected M. tuberculosis’s levels of magnesium, manganese, zinc and iron, but the only change observed in the total bacterial metal levels of M. tuberculosis under replicating conditions that correlated with the mycobactericidal effects of 4HQs was a fall in magnesium. In M. tuberculosis under nonreplicating conditions, the 4HQs had a significant effect on magnesium and manganese levels, but as will be discussed below, increased extracellular concentrations of these metals had little effect on the mycobactericidal actions of 4HQs under nonreplicating conditions (data not shown). These results pointed to differences in metallostasis in the two metabolic states and differences in the mechanism of action of the same 4HQs on M. tuberculosis in those states.

Total magnesium levels in replicating, DMSO-treated M. tuberculosis were significantly higher in M. tuberculosis bearing any of three different 4HQ resistance mutations than in WT M. tuberculosis, and the resistance-conferring mutations markedly blunted the fall in magnesium seen in response to treatment of M. tuberculosis with 2504 or 2178 (Fig. 4G; see Fig. S5A in the supplemental material). The differences were not due to changes in viable cell number over the 24 h of exposure, as the numbers of CFU/ml in each sample were comparable (Fig. 4H). In contrast, iron levels in the 6(D285G) and 3(E212D) mutants were elevated above WT in the DMSO-treated samples, but not in the 4(A317S) mutant, and the 4HQs had no effect on levels of iron in mutants (Fig. S5B). In nonreplicating M. tuberculosis, CorA mutations also blunted the 4HQ-induced fall in magnesium at a time when CFU counts were the same in each sample (Fig. S5C).

10.1128/mBio.01405-19.6 FIG S5 Magnesium levels in WT and CorA mutant strains under replicating and nonreplicating conditions. M. tuberculosis cells were exposed to compounds for 24 h under replicating conditions, and total contents of (A) magnesium and (B) iron were determined in each strain. Statistical significance in panel A was determined in comparison to the WT control in response to DMSO, 2504, or 2178. Statistical significance in panel B was determined in comparison to the WT DMSO control; there was no change in iron levels in response to compound exposure when each strain was compared to its own DMSO-treated control. Data are means ± SD from triplicates. *, *P* < 0.02; **, *P* < 0.002; ***, *P* < 0.0002. Results from WT and 3(E212D) cells were observed in two independent experiments, while experiments with 6(D285G) and 4(A317S) cells were conducted once. (C) Total magnesium contents and CFU/ml in WT and 3(E212D) cells exposed to either 2178 or 2504 for 48 h under nonreplicating conditions in the 4-stress model. Statistically significant differences between WT and mutant strains in response to treatment or vehicle were noted. Data are means ± SD from triplicates. *, *P* < 0.02; **, *P* < 0.002; ***, *P* < 0.0002. Results with WT cells are representative of two experiments, and results shown for 3(E212D) are from one experiment. (D) Concentration of free intracellular Mg^2+^ in replicating M. tuberculosis cells after 48 h of exposure to 2178 (15 μM) or SAR1 (15 μM). Values are the average percentage of the starting (time zero [T0]) concentration remaining. Download FIG S5, PDF file, 0.1 MB. Copyright © 2019 Lopez Quezada et al. 2019 Lopez Quezada et al. This content is distributed under the terms of the Creative Commons Attribution 4.0 International license.

These results supported the interpretation that mutations in CorA reduced the extent of 4QH-dependent magnesium depletion, and in doing so, helped mitigate the mycobactericidal effect of 4HQs. However, total cellular magnesium includes magnesium associated with the cells’ outer membrane, periplasm, inner membrane, ribosomes, and nucleoid, while the gating of the CorA channel is expected to be affected only by the levels of Mg^2+^ in the cytosol (unbound intrabacterial Mg^2+^). Therefore, we turned next to measurements of the free Mg^2+^ in cell-wall-depleted M. tuberculosis lysates. We used the activity of reagent glycerol kinase, a Mg^2+^-dependent enzyme, to report the levels of free Mg^2+^ in M. tuberculosis lysates. The assay’s limit of detection in our hands required the use of a dense cell culture to improve sensitivity. The assay cannot detect bound magnesium, such as intracellular magnesium bound to ribosomes and nucleic acids. Based on the total number of cells used to generate each control sample, the estimated soluble protein content per cell varied from 0.2 to 0.5 ng. Using the estimated amount of soluble protein per cell and the number of nmol of free Mg^2+^ per mg of protein in each sample, the average intracellular concentration of free Mg^2+^ in control M. tuberculosis cells after 24 h of incubation was estimated at ∼1 mM. Exposing replicating M. tuberculosis to 2178 at 25 μM caused the concentration of labile bacterial Mg^2+^ to drop to undetectable levels within 6 h (Fig. 4I). At the same concentration, 2504 caused a 43% drop in labile bacterial Mg^2+^ after 24 h and a 57% drop after 48 h. These changes correlated with the kill kinetics: 2178 reduced the CFU/ml by 3 log_10_ in 48 h, while the cidal effect of 2504 was weaker and slower (Fig. 4J). A 48-h treatment with SAR1 also resulted in free Mg^2+^ dropping to undetectable levels (Fig. S5D).

### Lack of Mg^2+^ chelation by 4HQs and SAR1 in aqueous media.

We considered the possibility that 4HQs and SAR1 chelate intrabacterial Mg^2+^ and promote its removal from the cell. Infrared spectroscopy studies in organic solvent detected a potential interaction of SAR1 with Mg^2+^ (see Fig. S6, inset table, in the supplemental material), based on the appearance of a water band (OH) at 3,389 cm^−1^ and of new bands at 441, 485, and 505 cm^−1^ in the far-infrared (far-IR) region. This absorption pattern in the far-IR spectrum of metal-ligand complexes has been noted previously (32). Additionally, shifts in the pyridine γ CH and all the carbonyl bands were observed (Fig. S6A and B). However, we detected no interaction between 2178 and Mg^2+^ (Fig. S6D and E), although cobalt appeared to complex with 2178 (Fig. S6F). No complexation was detected between 2504 and Mg^2+^ (Fig. S6G and H). Iron appeared to interact with SAR1 (Fig. S6C) and 2504 (Fig. S6I).

10.1128/mBio.01405-19.7 FIG S6 Infrared spectrum analysis of solid samples of compounds potentially combined with metals. (A to E) IR spectra of (A) SAR1 alone, (B) SAR1 and magnesium, (C) SAR1 and iron, (D) 2178 alone, and (E) 2178 and magnesium. (F) Far-IR spectra of 2178 and cobalt (red) compared to 2178 alone (blue). (G to I) IR spectra of (G) 2504 alone, (H) 2504 and magnesium, and (I) 2504 and iron. The inset table summarizes relevant observations. Download FIG S6, PDF file, 0.3 MB. Copyright © 2019 Lopez Quezada et al. 2019 Lopez Quezada et al. This content is distributed under the terms of the Creative Commons Attribution 4.0 International license.

**FIG 5:**
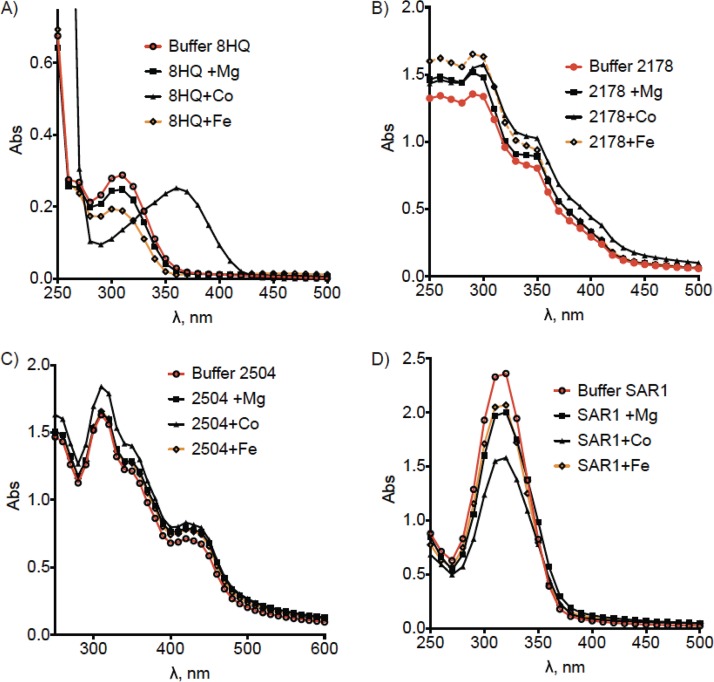
Tests of complexation in aqueous buffer. Shown is UV-vis analysis of (A) 8HQ, (B) 2178, (C) SAR1, or (D) 2504 incubated with various metals in saline HEPES buffer at pH 7.4. Results are from one experiment representative of three.

Moreover, UV-visible (UV-vis) spectroscopy failed to detect complexation of 4HQs with Mg^2+^. As a control, we confirmed that a complex of 8-HQ and cobalt ions in HEPES-saline buffer could be readily detected (Fig. 5A). There was no shift in the UV-vis absorption spectrum with 2178 or SAR1 or 2504 and Mg^2+^ in HEPES-saline buffer (Fig. 5B to D). Complexation between 2178 and various metals, including Mg^2+^, could not be detected by UV-vis spectroscopy in phosphate-buffered saline (PBS) or in sodium phosphate buffer at pH 6.6 or 5.0, the pH of replicating and nonreplicating media, respectively (data not shown). Although no shift in the absorbance spectrum was detected when SAR1 was incubated with Mg^2+^, Co^2+^, or Fe^2+^, there was a drop in the level of absorbance, particularly with Co^2+^. This could be due to the compound precipitating out of solution (Fig. 5D) rather than an indication of possible complexation, since no interaction between SAR1 and Co^2+^ was detected by IR spectroscopy (Fig. S6). Further studies would be needed to explain the apparent discrepancy between the IR and UV-vis spectral analyses with respect to SAR1.

In sum, while SAR1 may stably complex with Mg^2+^ in an organic solvent, 4HQs do not chelate Mg^2+^ either in an organic solvent or in aqueous media. We conclude that it is unlikely that 4HQ-dependent loss of intrabacterial Mg^2+^ and associated death of M. tuberculosis are caused by direct sequestration of metals by these compounds.

### Supplemental Mg^2+^ reduces the mycobactericidal effect of 4HQs.

Based on the results to this point, we hypothesized, first, that 4HQs and SAR1 kill M. tuberculosis by causing a loss of intrabacterial Mg^2+^ as a result of interaction with unknown targets, and second, that mutations in CorA that disfavor its closed conformation protect M. tuberculosis from the 4HQs and SAR1 by allowing a compensatory influx of Mg^2+^. If so, addition of supplemental Mg^2+^ to the growth medium might reduce the cidal effect of these compounds.

**FIG 6:**
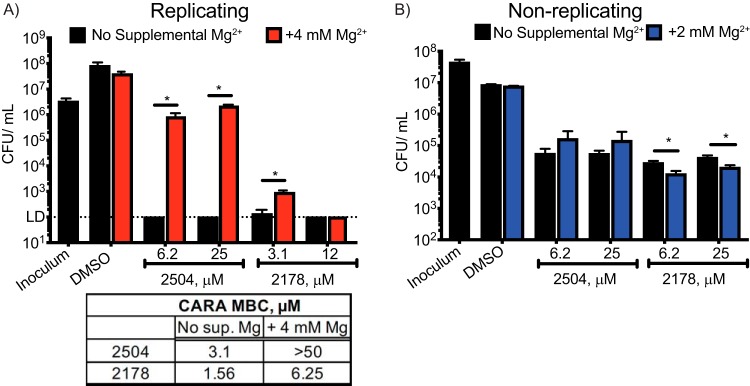
Reduction in cidality of 4HQs by supplementation of the medium with Mg^2+^. Minimal medium (MM) was supplemented with 4 mM MgCl_2_. Shown are changes in CFU/ml of (A) replicating and (B) nonreplicating M. tuberculosis exposed to compound 2504 or 2178. Data are means ± SE from one of two similar experiments, each in triplicate. *, *P < *0.02. The inset table shows the shift in CARA_MBC_ brought about by Mg^2+^ supplementation of the medium of replicating M. tuberculosis exposed to 2178 or 2504. Data are means from one of three similar experiments, each in triplicate.

Consistent with this prediction, replicating M. tuberculosis was protected from 25 μM 2504 as the concentration of Mg^2+^ in the minimal medium (approximately 300 μM Mg^2+^) was increased by an additional 4 mM (Fig. 6A). The impact on M. tuberculosis viability was striking. In the presence of 4 mM supplemental MgCl_2_, the activity of 2504 at 25 μM was reduced from >5 log_10_ of killing to <2 log_10_, while the cidality of 2178 was reduced by 1 log at 3.1 μM (Fig. 6A). In contrast, increasing the concentrations of either Mn^2+^ or Fe^2+^ had no protective effect, nor did the latter metals influence the effect of supplemental Mg^2+^ on the cidality of 2504 (see Fig. S7A in the supplemental material). Supplementation with Co^2+^ had an effect opposite to that of Mg^2+^, increasing the sensitivity of M. tuberculosis to 2504 and 2178 (Fig. S7B), while Ni^2+^ had no effect (Fig. S7C). However, supplemental Mg^2+^ had no effect on the activity of 2504 on nonreplicating M. tuberculosis (Fig. 6B) and caused a slight (<0.5 log_10_) but statistically significant increase in the sensitivity of nonreplicating M. tuberculosis to 2178 (Fig. 6B).

10.1128/mBio.01405-19.8 FIG S7 Metal supplementation of growth media. (A) A checkerboard assay was executed to test the effect of various metal concentrations on the inhibitory activity of 2504 at 25 μM on replicating M. tuberculosis. Complete minimal medium was supplemented with additional Mg^2+^ and Mn^2+^ at the indicated concentrations and at a final Fe^2+^ concentration of 50 or 10 μM. (B and C) The single metal supplementation effect of 100 μM of CoCl_2_ or 500 μM of NiCl_2_ on the activity of (B) 2504 or (C) 2178. (D) SAR1 activity in response to excess magnesium in complete minimal medium as compared to (E) rifampin (Rif). (F) Effect of increasing concentrations of Mg^2+^ on the IC_90_ of BCG cells in response to SAR1 exposure. Data are means ± SE from one experiment representative of two independent experiments. Download FIG S7, PDF file, 0.2 MB. Copyright © 2019 Lopez Quezada et al. 2019 Lopez Quezada et al. This content is distributed under the terms of the Creative Commons Attribution 4.0 International license.

When we varied the concentrations of 4HQ or BA derivative and held supplemental MgCl_2_ constant at 4 mM, the supplemental Mg^2+^ increased the CARA_MBC_ by 4-fold for 2178 and by >16-fold for 2504 (Fig. 6A, inset table) and SAR1 (Fig. S7D), while extra Mg^2+^ had no effect on the response to rifampin (Fig. S7E).

Results were comparable with SAR1 and BCG. Increasing extracellular Mg^2+^ antagonized the activity of SAR1 against BCG and further increased the SAR1 resistance of the BCG CorA 1(A317S) mutant strain (Fig. S7F).

## DISCUSSION

Nutritional immunity is a term used to describe a host-mediated impairment of availability of nutrients or micronutrients. The term often refers to iron sequestration away from a pathogen-containing phagosome (33, 34) but can also refer to sequestration of zinc or manganese (35). Similarly, a number of exogenous compounds explored for their antimicrobial potential have been found to disrupt bacterial metallostasis, but where the mechanism has been uncovered, it usually involves chelation. For example, dithiolopyrrolones disrupt zinc metallostasis in E. coli not by changing the total zinc content of cells but rather by acting as zinc chelators (36), and a pyrazolopyrimidininone kills M. tuberculosis by acting as an intrabacterial iron chelator (37). Calcimycin (A23187) is an ionophore of divalent cations (38) that, in combination with polymyxin, induced the efflux of intracellular free magnesium by altering the permeability of the cytoplasmic membrane of E. coli (39). To our knowledge, the present results are the first to identify depletion of bacterial magnesium as the mechanism of action of nonchelating antibacterial compounds.

These results highlight the basic scientific interest and potential translational value of studying bacterial metallostasis. Our findings also call attention to the dearth of quantitative measurements of metals in mycobacteria and the difficulty in predicting the directions of metal transport, given the changing demands of maintaining metallostasis in different metabolic settings. In E. coli, intrabacterial Mg^2+^ levels were found to equilibrate with extracellular levels over 4 orders of magnitude (40). In contrast, Goodsmith et al. found that replicating M. tuberculosis cells maintained the same total magnesium content after 6 days of incubation in minimal medium that contained either 2 mM Mg^2+^ or 0.25 mM Mg^2+^ (28). Although we cannot compare the per-cell magnesium levels of M. tuberculosis in the earlier study to those seen here with the same ICP-MS technique, the results of Goodsmith et al. indicate that M. tuberculosis is able to preserve a steady total magnesium content in the face of marked changes in extracellular Mg^2+^concentration.

Mg^2+^ circulates in human plasma at 0.7 to 1.0 mM, more than twice the 430 μM found in 7H9 broth and 300 μM in minimal medium. Levels of Mg^2+^ in the extracellular fluid of tubercular pneumonitic zones, granulomas, caseum, or cavities have not been reported, to our knowledge. Our estimates of magnesium content per M. tuberculosis cell growing in 7H9 broth (∼60 mM total magnesium; ∼1 mM free Mg^2+^) demonstrate that M. tuberculosis *in vitro* can maintain a differential between extracellular and intrabacterial levels. Macrophage phagosomes containing ingested Salmonella enterica were inferred to have levels of Mg^2+^ of 1 mM (41), while Mg^2+^ concentrations in epithelial cell vacuoles containing S. enterica were estimated to be between 10 and 50 μM (42). There are apparently no such data for mycobacterium-containing phagosomes. Levels of several metals fell in phagocytized mycobacteria compared to those in standard broth culture (43), but Mg^2+^ levels were not measured in that study. Nonetheless, genetic evidence from the knockout of putative metal transporters implicates magnesium homeostasis as critical for mycobacterial survival in the host (44, 45). Consistent with that inference, Goodsmith et al. (31) found that M. tuberculosis lacking a gene they named *perM* was attenuated in the chronic phase of infection in mice. The only phenotype of *perM-*deficient M. tuberculosis that the authors could identify *in vitro* that might explain the strain’s attenuation was its inability to grow in media with <100 μM Mg^2+^. This may signify that M. tuberculosis’s main niche in the chronic phase of infection in the mouse model studied—that is, the macrophage phagosome—is a low-magnesium environment (28), in which disruption of M. tuberculosis’s ability to maintain Mg^2+^ homeostasis could be mycobactericidal. Our demonstration of the cidal effect of 4HQ- and SAR1-induced Mg^2+^ depletion confirms that M. tuberculosis cannot survive unless it can defend its levels of Mg^2+^ from depletion.

The potential consequences of Mg^2+^ depletion are multifold, given the number of Mg^2+^-dependent enzymes and the role of Mg^2+^ as a counter ion for neutralizing the negative charge of several biomolecules, including several membrane macromolecules. The loss of cytosolic Mg^2+^ would have a particularly negative impact on ribosome assembly and protein synthesis (46), which could impact both replicating and nonreplicating M. tuberculosis (8, 47). In E. coli and *Salmonella*, in a low-magnesium environment, ribosome function is maintained by upregulating magnesium transport channels and damping translation rates (48). In Bacillus subtilis, increased uptake of Mg^2+^ in individual cells correlated with phenotypic tolerance for ribosome-targeting antibiotics (49). This raises the possibility that magnesium-depleting compounds could synergize with ribosome-targeting antibiotics. Loss of membrane-bound magnesium could pose an earlier and even greater threat to cell viability than impairment of protein synthesis. In short, the relationship between Mg^2+^ depletion and mycobacterial cell death is likely to be complex.

CorA, MgtE, and MgtA/B constitute the three known classes of magnesium transporters in bacteria. Although all can carry out magnesium import, they differ in structure, their response to other cations, and their regulatory mechanisms. It has been postulated that bacteria express more than one class of magnesium transporter in order to respond adequately to changing growth conditions (50). The CorA and MgtE channels are the most widely distributed housekeeping Mg^2+^ transporters and are constitutively expressed. They import magnesium via an electrochemical gradient (51). The cytoplasmic domains of CorA and MgtE contain multiple magnesium binding sites; upon Mg^2+^ binding to those sites, the channel undergoes a major conformational change and loss of its uptake function (52, 53). In this way, a bacterium can regulate how much Mg^2+^ is within the cell. However, channels can also mediate efflux and could potentially be induced by the compounds to export magnesium out of the cell. In S. enterica (54) and E. coli (55), CorA homologs appeared to be more responsible for mediating Mg^+2^ efflux than MgtA or MgtB. However, neither transposon-mediated disruption of the channels nor HexaCo inhibition of CorA substantially affected the efficacy of SAR1 or 4HQs, respectively. In fact, the channels appeared to mitigate the compound’s activity since their loss led to an increase in sensitivity in M. tuberculosis, and overexpression of MgtE in BCG protected the cells from the activity of SAR1. Why overexpression of WT CorA did not have a similar effect is not clear, considering that depletion of cytoplasmic Mg^2+^ upon compound exposure would favor the open conformation of the channel.

Other potential metal efflux mechanisms have been reported in M. tuberculosis, presumably serving to prevent metal intoxication (56, 57). Several P-ATPase efflux pumps have been implicated in assisting M. tuberculosis cells in preventing a buildup of Zn^2+^ (58), Co^2+^ (59), and Cu^+^ (60). These pumps could potentially mediate the efflux of magnesium, as there are few data on the specificity of these channels.

A nuclear magnetic resonance (NMR) study suggested that the transmembrane domain of M. tuberculosis CorA can bind Mg^2+^ (27), but there is apparently no biophysical evidence for CorA’s role in Mg^2+^ transport in M. tuberculosis. We hypothesize that CorA in M. tuberculosis militates against the effects of 4HQs and SAR1 by supporting Mg^2+^ influx and that the resistance-conferring mutations promote that activity. However, mutational analysis of the TmCorA could shed some light on effects of the mutations found in M. tuberculosis CorA. Mutations intended to loosen coil-coil interactions of the TmCorA, including salt bridge formation between K223 and D263 in the closed conformation, “disfavored” the closed conformation of the channel (61). These mutations are similar to the predicted location and nature of the N285D mycobacterial mutations and, to a lesser extent, the L229V mutation observed in BCG and M. tuberculosis, in which the disruption of the intrahelical packing would favor the more disordered conformation observed in the open cryo-electron microscopy (cryo-EM) structures of TmCorA (62). Furthermore, according to MD simulations of the central part of the TM channel, as a potential result of the new hydrogen-bond pattern introduced by the mutated A317S, the internal diameter of the channel was predicted to be higher for the mutated CorA than for the wild-type protein. For the mutated protein, the polar ring created by the side-chain oxygen atoms of the five serines modifies the hydrophobicity of the channel in its central part. Both events could lead to shift the ground state of CorA toward a state that favors the transfer of Mg ions through the protein. This predicted instability of the closed conformation in the CorA mutants or the increased permeability of the channel would explain why the mutants exhibited higher levels of total magnesium with or without treatment with 4HQs or SAR1 under both replicating and nonreplicating conditions.

In structure-activity relationship studies not reported here, we were unable to overcome adequately the liabilities of the 4HQ and BA derivatives, such as their poor solubility and cytotoxicity. Nevertheless, their value has been multifold. First, they illustrate the vulnerability of Mg^2+^ metallostasis in mycobacteria. It remains for further studies to identify the primary targets responsible for this effect. Second, they demonstrate that one and the same compound can kill replicating and nonreplicating M. tuberculosis by different mechanisms. For example, 2178 rapidly depleted Mg^2+^ in replicating M. tuberculosis, but nonreplicating M. tuberculosis sustained its Mg^2+^ even after 48 h of treatment with 2178. Moreover, resistant clones that were selected under replicating conditions, all of which had mutations in CorA, were not protected under nonreplicating conditions.

Our attempts to isolate resistant mutants under replicating conditions did not result in the identification of target(s). This could be due to the existence of multiple targets, such that a single clone with protective mutations in each target could be too rare to detect in populations of the size we were able to study. However, we did succeed in selecting resistant clones under nonreplicating conditions that had CARA_MBC_ values against 4HQs 4- to 8-fold higher than those for WT cells and that were resistant only under nonreplicating conditions. Remarkably, whole-genome resequencing of 4 such clones revealed no discernible SNPs, deletions, translocations, or copy number variations (data not shown). As discussed elsewhere, the occasional inability of genome resequencing to offer candidate explanations for the phenotypes of bacterial clones invites consideration of epigenetic control (63). Such observations underscore the importance of better characterizing the metabolic differences and dependencies of M. tuberculosis under different environmental conditions and replicative states.

## MATERIALS AND METHODS

### Compounds.

Compounds 2178, 2504, and 2150 were initially provided by Sanofi (Paris, France) for screening and initial experiments. The compounds were then resynthesized and purified at the University of North Carolina, Chapel Hill. Sources of other compounds are listed in Text S1 in the supplemental material. SAR1, TMC207, and cyclohexyl-griselimycin were provided by Sanofi (Paris, France). 8-Hydroxyquinoline (8-HQ), activated charcoal, sodium resazurin, hexaammine cobalt chloride (HexaCo), isoniazid, and rifampin (Rif) were from Sigma-Aldrich, USA, and moxifloxacin hydrochloride (Moxi) was from Santa Cruz Biotechnology, USA.

10.1128/mBio.01405-19.1 TEXT S1 Supplemental methods. Download Text S1, PDF file, 0.1 MB. Copyright © 2019 Lopez Quezada et al. 2019 Lopez Quezada et al. This content is distributed under the terms of the Creative Commons Attribution 4.0 International license.

### Bacterial strains and culture methods.

Wild-type M. tuberculosis strain H37Rv (ATCC 25618) was grown under standard replicating conditions as described in Text S1. Two nonreplicating models were used in this study. The 4-stress model has been characterized (18) and used to discover antimycobacterials active against nonreplicating M. tuberculosis (64, 65) and is detailed in Text S1. Where indicated, we used the nutrient starvation (Loebel) model of nonreplication (66). After a month of starvation, cells were exposed to compounds for 7 days, after which cell survival was assessed.

### MIC, CARA, and generation of resistant mutants.

Details are given in Text S1. For M. tuberculosis, the MIC was the lowest concentration of compound that inhibited >90% of the cell growth in the vehicle control wells. For BCG, the MIC was the lowest concentration effecting ≥80% reduction in alamarBlue fluorescence relative to the signal for the no-drug control. The CARA_MBC_ was the lowest concentration of compound that reduced fluorescence to ≤1% of that in DMSO control wells, which corresponds to a difference in viable cell number of ≥2 log_10_ (67).

### Homology modeling and MD simulation of M. tuberculosis CorA.

Primary sequence alignments were generated using ClustalW. Three-dimensional (3D) homology models for M. tuberculosis CorA were built using the Thermotoga maritima CorA (TmCorA) crystal structures as templates. Using Molecular Operating Environment 2018.10 (MOE) software, T. maritima PDB 4i0u was used to model M. tuberculosis CorA in the closed conformation, and the open conformation of the CorA pentamer was modeled using T. maritima PDB 3jcg and 3jch. MD simulations were carried out as described in Text S1.

### Total metal ion counts using ICP-MS.

For analysis of replicating cells, cultures were washed and resuspended to an optical density at 580 nm (OD_580_) of 0.8 in a 3.5-ml/flask. A 25 μM concentration of either 685B, 2504, or 2178 was added to cultures using DMSO as the vehicle control. Samples collected at the beginning of the experiment were designated as “T0.” For experiments using the 4-stress model of nonreplication, cells were diluted to an OD_580_ of 0.9 with 3 ml/flask, preadapted to nonreplicating conditions for 24 h, and exposed to each compound at 25 μM for 24 and 48 h. CFU were taken at the time of collection. ODs were taken before harvesting the cells to ensure pellets were of similar mass. Cell pellets were stored at −80°C. Prior to shipment to Agilent Technologies, cell pellets were heat killed by incubation at 85°C for 1 h. Cell were dissolved in 70% nitric acid then incubated at 85°C for 2 h. PBS was then added to ship the samples in 35% nitric acid. Elements were quantified on an Agilent 7800 ICP-MS device equipped with an x-lens, SPS-4 autosampler, MicroMist nebulizer, and the HMI interface as described in Text S1.

### Quantification of intracellular free magnesium pool.

Cells were diluted to an OD_580_ of 0.8 and compound (or DMSO) was added to the samples to a final concentration of 25 μM. At the indicated times, cells were centrifuged, washed in PBS plus tyloxapol (Tyl), and stored at −80°C until assay with a magnesium assay kit (Sigma, USA). Cells were resuspended in 600 μl of Mg assay buffer and bead beaten to lyse the cells. Cellular debris and unlysed cells were removed by centrifugation. Lysates were filtered and assayed according to the manufacturer’s instructions. Samples (50 μl) were used without dilution.

### Compound-metal complexation assays.

HEPES buffer (50 mM) was prepared with 150 mM NaCl, and the pH was adjusted to 7.0. Sodium phosphate buffer (SPB) (50 mM) was adjusted to pH 6.6 (the approximate pH of 7H9 medium) or pH 5.0 (the pH of NR medium). Metal and compound were mixed 1:1 at either 50 or 100 μM in SPB, saline HEPES buffer, PBS, PBS with glycerol, or 7H9 medium. 8-Hydroxyquinoline, a well-documented metal chelator (19, 68, 69), was used as a control.

### Metal supplementation of minimal medium.

To control metal content of the medium, we prepared minimal medium (MM) as described previously (70). One liter of basal (incomplete) MM contained 20 ml glycerol, 5 g asparagine, 5 g KH_2_PO_4_, and 0.02% tyloxapol. The pH was adjusted to 7.0. Metals were removed from the medium and the albumin-dextrose-NaCl (ADN) supplement by adding Chelex (Bio-Rad) overnight at 4°C and repeating the following day. Separate 1,000× solutions were made consisting of 3.66 mM ZnCl_2_, 0.66 mM MnSO_4_, 333 mM MgSO_4_, and 50 mM FeCl_3_. Complete MM was prepared the day of the experiment by adding 100 ml ADN and 1 ml each of ZnCl_2_, MnSO_4_, and MgSO_4_ to 1 liter of basal MM. Unless otherwise specified, MM containing 50 μM FeCl_3_ was used. For testing iron dependency of activity, cells were preadapted for 20 days in complete MM with 2 μM FeCl_3_, and compound susceptibility was tested in either 50 or 2 μM. Additional magnesium, manganese, cobalt, or nickel was added as needed for experiments testing the effects of metal supplementation on bacterial susceptibility to compounds. MICs and CARAs were carried out as before.

### Statistical analysis.

Comparisons were analyzed by a two-tailed Student's *t* test with GraphPad Prism 8. Values of *P* < 0.02 were considered significant. CARA assay results are presented as means ± standard error (SE), while other data are means ± standard deviation (SD). Unless otherwise specified, experiments were carried out in triplicate. Each result illustrated is representative of results from 2 or more independent experiments, as indicated in the figure legends.
