# Supplementary material for: Bactericidal Disruption of Magnesium Metallostasis in Mycobacterium tuberculosis Is Counteracted by Mutations in the Metal Ion Transporter CorA
Source: mBio. 2019 Jul 9;10(4):e01405-19. doi: 10.1128/mBio.01405-19 (PMC6747715; doi:10.1128/mBio.01405-19)
Supplement: FIG S1 [file mBio.01405-19-sf001.pdf]

A)

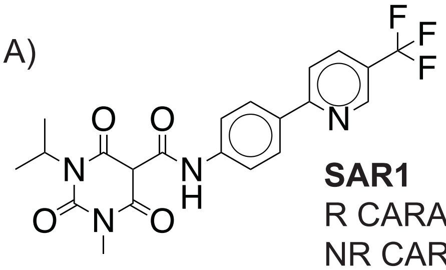

SAR1

R $CARA_{MBC}$: 0.78 μM

NR $CARA_{MBC}$: 0.78 μM

HepG2 LD_{50}: 3 μM

M. bovis BCG

IC_{80}: 0.4 μM

B)

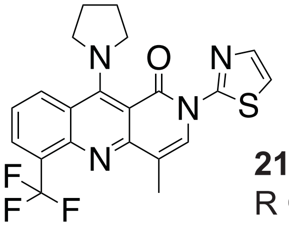

2150

R $CARA_{MBC}$: 6.2 μM

NR $CARA_{MBC}$: 12 μM

HepG2 LD_{50}: 7.0 μM

Fig. S1. Structure, antimycobacterial activity and cytotoxicity of SAR1 and 4HQ 2150. (A) SAR1 and (B) 2150 were tested against Mtb under replicating (R) or non-replicating (NR) conditions in the 4-stress model and against human HepG2 cells. The activity of SAR1 against replicating *M. bovis* BCG was determined by IC_{80}. Data are means from one of two similar experiments, each in triplicate.
