# Supplementary material for: Bactericidal Disruption of Magnesium Metallostasis in Mycobacterium tuberculosis Is Counteracted by Mutations in the Metal Ion Transporter CorA
Source: mBio. 2019 Jul 9;10(4):e01405-19. doi: 10.1128/mBio.01405-19 (PMC6747715; doi:10.1128/mBio.01405-19)
Supplement: TABLE S1 [file mBio.01405-19-st001.docx]

**Table S1. Selective resistance to SAR1 by BCG *corA* mutants.**

| Selection compound | Clone | AA change from *corA* SNPs | IC_80_, μM  (replicating conditions) | | | |
| --- | --- | --- | --- | --- | --- | --- |
|  |  |  | SAR1 | INH | TMC207 | Cyclohexyl-griselimycin |
| SAR1 | 1 | A317S | 2.8 | 0.6 | 0.4 | 0.07 |
|  | 2 | A317S | 2.5 | NT | 0.4 | 0.36 |
|  | 3 | L229V | 2.3 | 0.2 | 0. | 0.06 |
|  | 4 | G299S | 1.7 | 1.0 | 0.3 | 0.09 |
| None (WT) | BCG | -- | 0.20 | 0.34 | 0.23 | 0.02 |
