# Supplementary material for: Bactericidal Disruption of Magnesium Metallostasis in Mycobacterium tuberculosis Is Counteracted by Mutations in the Metal Ion Transporter CorA
Source: mBio. 2019 Jul 9;10(4):e01405-19. doi: 10.1128/mBio.01405-19 (PMC6747715; doi:10.1128/mBio.01405-19)
Supplement: FIG S2 [file mBio.01405-19-sf002.pdf]

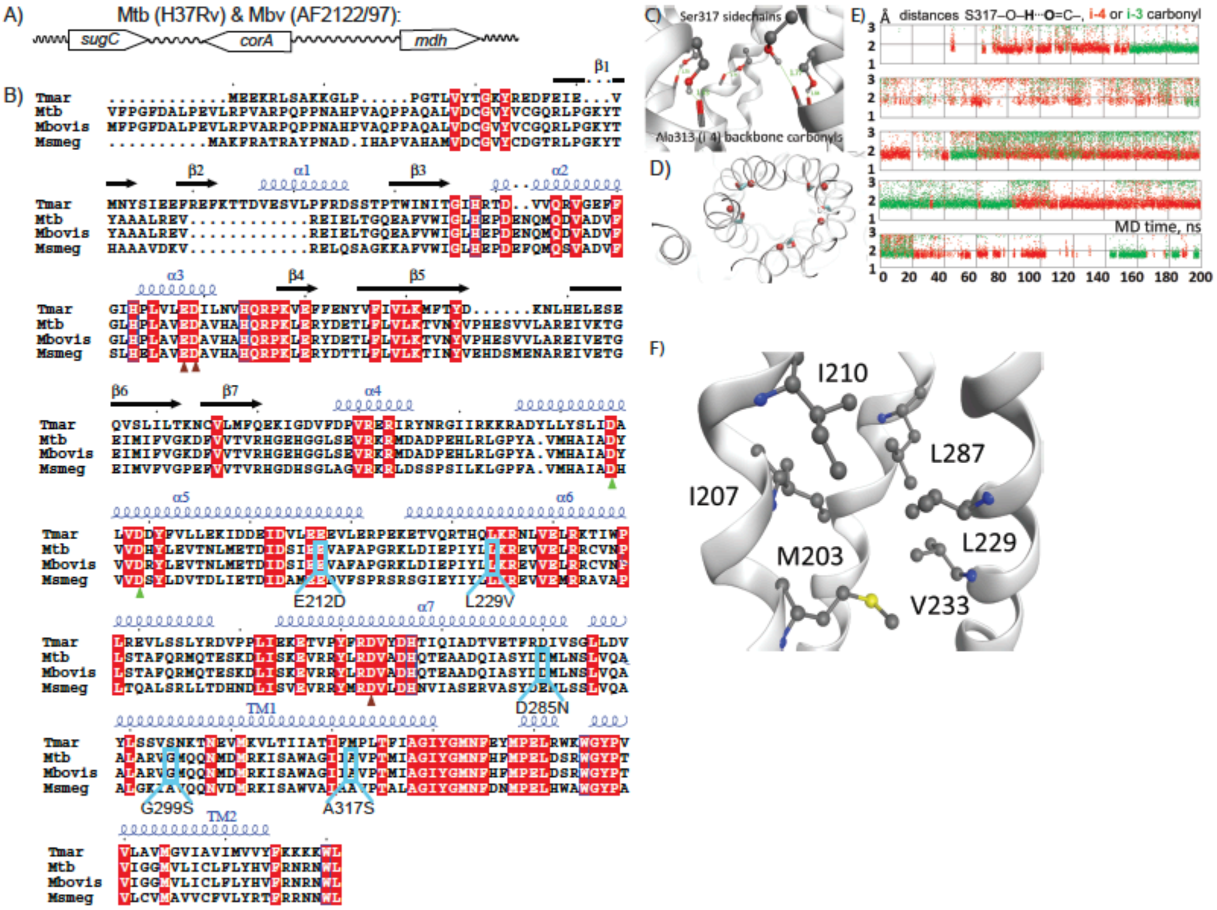

Fig. S2. Homology modeling of the mycobacterial CorAs with the CorA from *T. maritima* (Tm). (A) Position of *corA* in the *M. bovis* and Mtb genome. (B) Primary amino acid sequence alignment of *T. maritima* (Tm), Mtb, *M. bovis*, and *M. smegmatis* CorA. Mutations in resistant clones are in blue. Green triangles mark metal binding site M1; burgundy triangles mark cytoplasmic metal binding site M2. (C) MD analysis of the Ser317 sidechain H-bonds in the five CorA monomers. Orientation of the Ser317 sidechains in the CorA channel extracted from a 100ns MD trajectory snapshot revealed H-bonds with i-4 Ala313 backbone carbonyl oxygen. (D) The same frame as in C viewed from the intracellular part of the protein, Ala313 carbonyl oxygen atoms are depicted as red spheres. (E) Frequency of the hydrogen bond formation between the hydroxyl of S317 and the carbonyl oxygen atom (S317–O–**H**···**O**=C–) at position i-4 (Ala313, in red dots) and i-3 (Gly314, in green dots), during 200 ns MD simulation.

Distances vary between 1.7 and 2.1Å, approximately. (F) Mapping of the L229 location on the model of Mtb CorA using the closed conformation of the TmCorA structure in PDB 4I0U.
