# Supplementary material for: Bactericidal Disruption of Magnesium Metallostasis in Mycobacterium tuberculosis Is Counteracted by Mutations in the Metal Ion Transporter CorA
Source: mBio. 2019 Jul 9;10(4):e01405-19. doi: 10.1128/mBio.01405-19 (PMC6747715; doi:10.1128/mBio.01405-19)
Supplement: FIG S3 [file mBio.01405-19-sf003.pdf]

A)

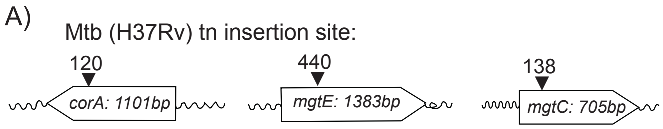

B)

Sensitivity of transposon mutants of metal transporters

	CARA MBC (μM)			
	2504	2178	SAR1	Rif
WT	3.1	3.1	0.78	0.25
corA::tn	1.56	1.56	0.39	0.25
mgtC::tn	3.1	3.1	0.78	0.25
mgtE::tn	1.56	3.1	0.39	0.25

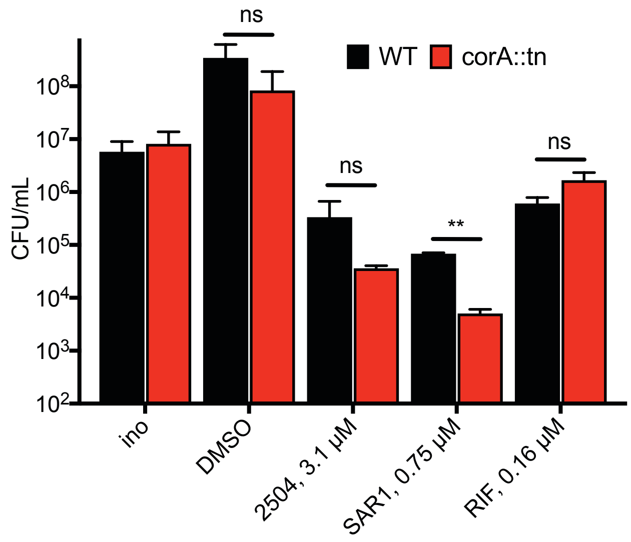

C)

D)

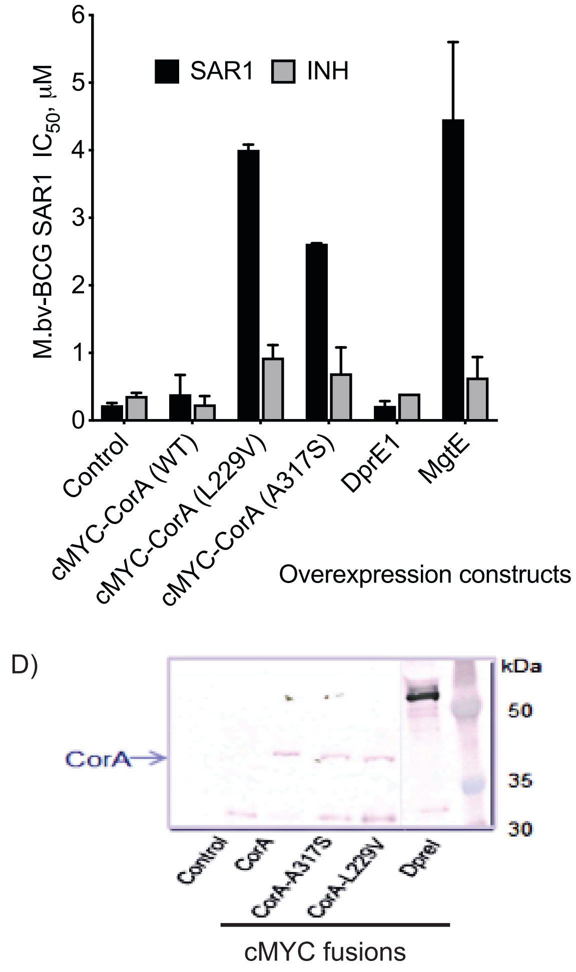

Fig. S3. Sensitivity of transgenic Mtb or BCG to compounds. (A) Transposon insertion sites (triangle) and the number of nt from the start site where the insertion cassette begins. (B) Sensitivity of transposon mutants to the indicated compounds. Impact of *corA* disruption on CFU/mL in response to 7 days of exposure to 2504, SAR1, and rifampicin. Data are means ± SD from one of two similar experiments, each in triplicate. Transposon mutants of Mtb *corA::tn*, *mgtE::tn*, and *mgtC::tn* H37Rv, a generous gift from Dr. Deborah Hung (AK Barczak, et. al., PLoS Pathog 13 (5), 2017), were supplemented with 25 μg/mL kanamycin. (C) IC_{50} of SAR1 in BCG expressing cMYC-tagged WT CorA or the mutant form of CorA. Known ion channel, MgtE, was also overexpressed and DprE served as a negative control. (D) Western blot indicating levels of cMYC-CorA fusion protein in BCG ectopically expressing

different CorAs. To express these proteins in BCG, the salicylate inducible promoter (gene Rv0560c) was PCR amplified and inserted between the NotI and EcoRI sites of pSC859, a shuttle vector containing the origins of replication for *E. coli* and *M. tuberculosis* and kanamycin resistance as a selection marker, kindly provided by Dr. Evelyne Liauzun. The fusion cMYC-CorA, cMYC-MgtE and cMYC-DprEI were PCR amplified and cloned under the control of the salicylate promoter between the NdeI and HindIII sites.
