# Supplementary material for: Bactericidal Disruption of Magnesium Metallostasis in Mycobacterium tuberculosis Is Counteracted by Mutations in the Metal Ion Transporter CorA
Source: mBio. 2019 Jul 9;10(4):e01405-19. doi: 10.1128/mBio.01405-19 (PMC6747715; doi:10.1128/mBio.01405-19)
Supplement: FIG S4 [file mBio.01405-19-sf004.pdf]

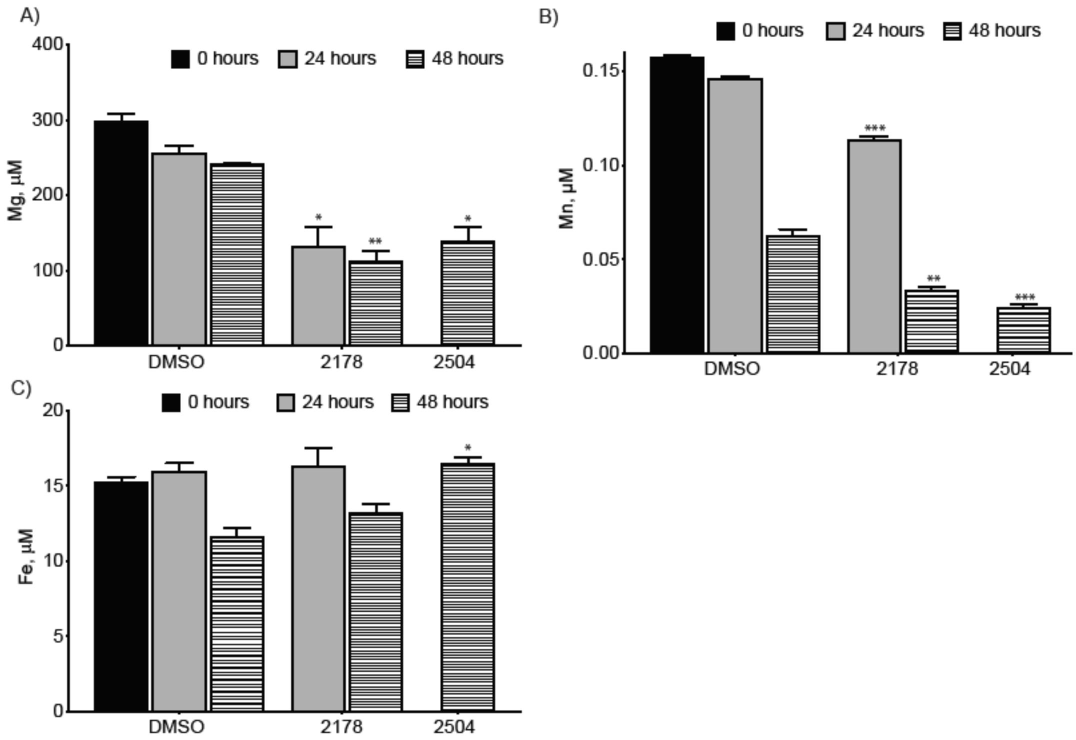

Fig. S4. ICP-MS analysis of metals in non-replicating cell exposed to 4HQs. Using the 4-stress model, non-replicating Mtb cells were exposed to 2504 or 2178 for 24 and 48 hours and the total contents of (A) magnesium, (B) manganese, and (C) iron were measured. Comparisons were made between the DMSO controls and the compound treated cells at the corresponding time points; *p<0.002, **p<0.0002, ***p<0.00002. Data are means ± SD of triplicates. 48 hour experiments are representative of two similar experiments, while the 24 hour experiment was conducted once.
