# Supplementary material for: Bactericidal Disruption of Magnesium Metallostasis in Mycobacterium tuberculosis Is Counteracted by Mutations in the Metal Ion Transporter CorA
Source: mBio. 2019 Jul 9;10(4):e01405-19. doi: 10.1128/mBio.01405-19 (PMC6747715; doi:10.1128/mBio.01405-19)
Supplement: FIG S5 [file mBio.01405-19-sf005.pdf]

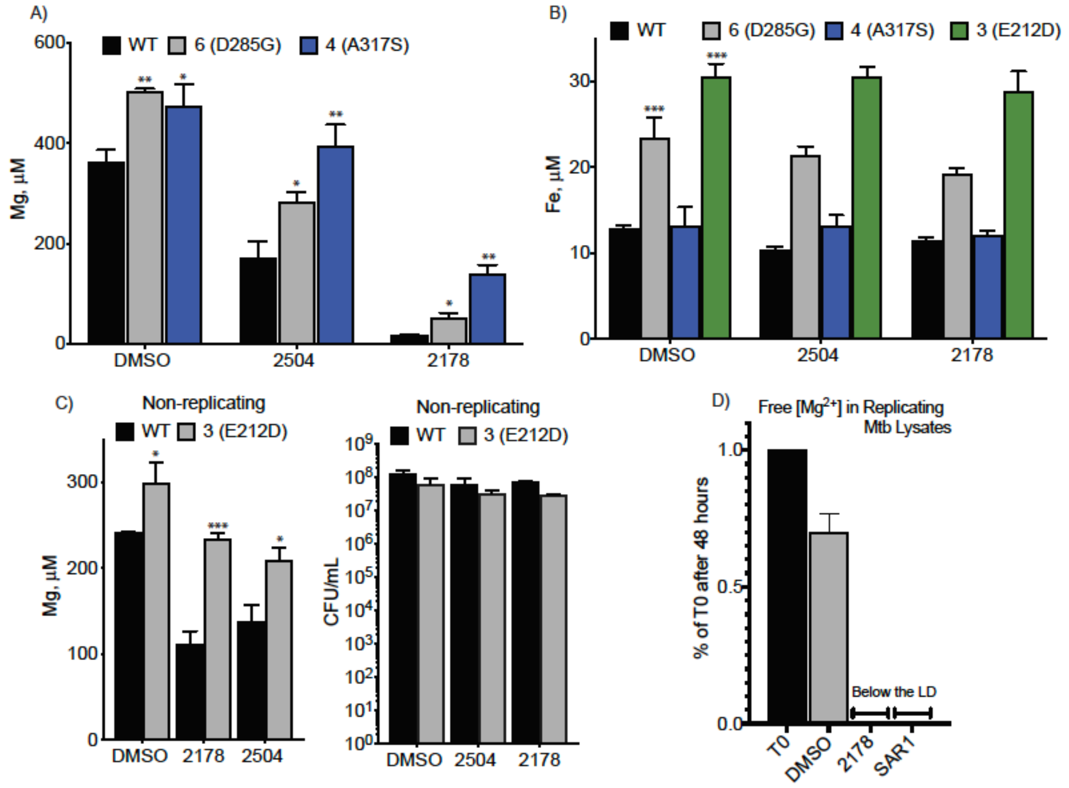

Fig S5. Magnesium levels in WT and CorA mutant strains under replicating and non-replicating conditions. Mtb cells were exposed to compounds for 48 hours under replicating conditions and total contents of (A) magnesium and (B) iron were determined in each strain. Statistical significance in (A) was determined in comparison to the WT control in response to DMSO, 2504, or 2178. Statistical significance in (B) was determined in comparison to the WT DMSO control; there was no change in iron levels in response to compound exposure when each strain was compared to its own DMSO-treated control. Data are means ± SD of triplicates. *p<0.02, **p<0.002, ***p<0.0002. Results from WT and 3 (E212D) cells were observed in two independent experiments while experiments with 6 (D285G) and 4 (A317S) were conducted once. (C) Total magnesium contents and CFU/mL in WT and 3 (E212D) cells exposed to either 2178 or 2504 for 48 hours under non-replicating conditions in the 4-stress model. Statistically significant differences between WT and mutant strains in response to treatment or vehicle were noted. Data are means ± SD of triplicates. *p<0.02, **p<0.002,

***p<0.0002. Results with WT cells are representative of two experiments and results shown for 3 (E212D) are from one experiment. (D) Concentration of free intracellular Mg^{2+} in replicating Mtb after 48 hours of exposure to 2178 (15 μM) or SAR1 (15 μM). Values are the average percent of the starting (T0) concentration remaining.
