# Supplementary material for: Bactericidal Disruption of Magnesium Metallostasis in Mycobacterium tuberculosis Is Counteracted by Mutations in the Metal Ion Transporter CorA
Source: mBio. 2019 Jul 9;10(4):e01405-19. doi: 10.1128/mBio.01405-19 (PMC6747715; doi:10.1128/mBio.01405-19)
Supplement: FIG S6 [file mBio.01405-19-sf006.pdf]

Summary		Infrared Spectrum Range		
Ligand/ Compound	Metal	Medium	Far	Observations
2178	Co	modified	modified	• Weak intensity υC-H bands shifted to 3137 and 3108 cm-1.<br>• The wide band between 2500 and 3000 cm-1 (υOH and υNH) is still visible.<br>• Band at 1585 cm-1 is more intense.<br>• Thiazole bands shifted towards Far IR, with apparition of new bands (224, 265, 310, 483 and 548 cm-1)<br>• Weak intensity υC-H bands shifted to 3137 and 3108 cm-1.<br>• The wide band between 2500 and 3000 cm-1 (υOH and υNH) is still visible.<br>• No carbonyls band-shift<br>• Band at 1585 cm-1 is more intense.<br>• Thiazole bands shifted towards Far IR, with apparition of new bands (224, 265, 310, 483 and 548 cm-1)
2178	Mg	unchanged	unchanged	
SAR1	Co	unchanged	unchanged	
SAR1	Mg	modified	modified	• Appartion of a water band (υOH) at 3389 cm-1.<br>• Carbonyl bands all shifted.<br>• Pyridine γ CH also shifted<br>• Apparition of bands at 325, 350, 441, 485 and 505 cm-1 in the Far IR region.
SAR1	Ni	unchanged	unchanged	
SAR1	Fe	modified	modified	• NH elongation is still visible.<br>• Carbonyl band shift from 1716cm-1 to 1708 cm-1.<br>• No shift of pyridine γ CH<br>• Apparition of bands at 313, 406, 490 et 530 cm-1 in the Far IR region.
2504	Mn	unchanged	unchanged	
2504	Mg	unchanged	unchanged	
2504	Fe	modified	modified	• Bands at 3115 and 3080 cm-1 shifted to 3100 and 3080 cm-1.<br>• Band at 2799 cm-1 disappeared.<br>• υC=C and υC=N thiazole bands also shifted.<br>• Bands apparition at 234, 283, 404, 465, 486 and 558 cm-1 in the Far IR region
		Compared to compoound alone		

A)

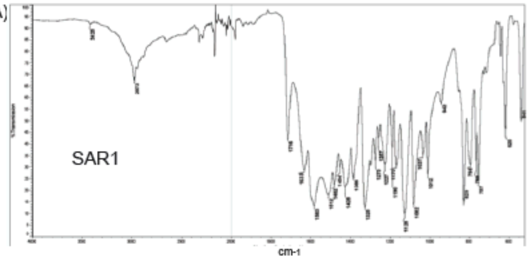

B)

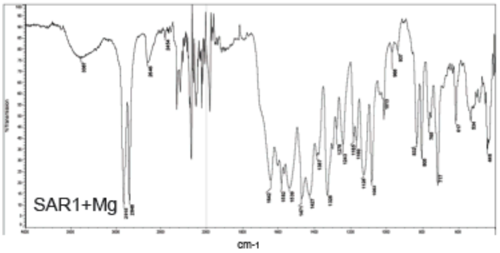

C)

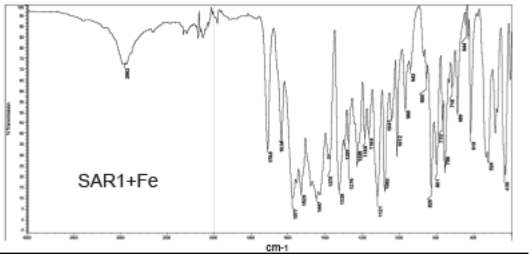

D)

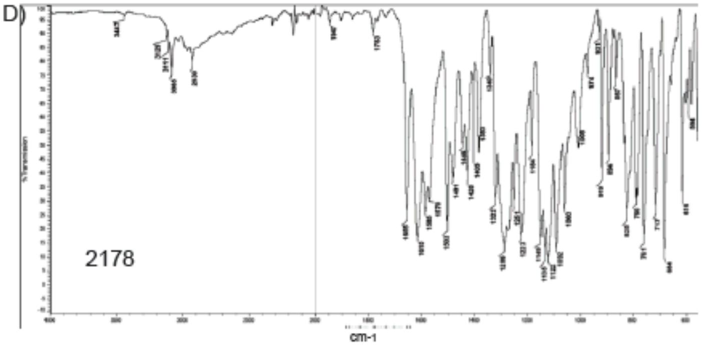

E)

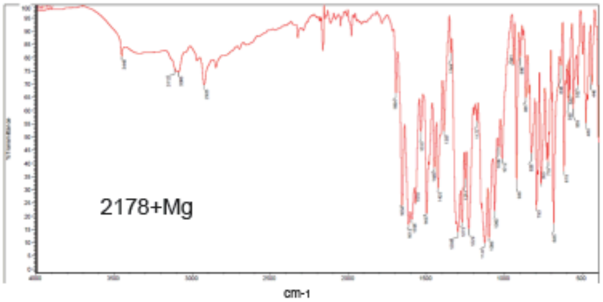

F)

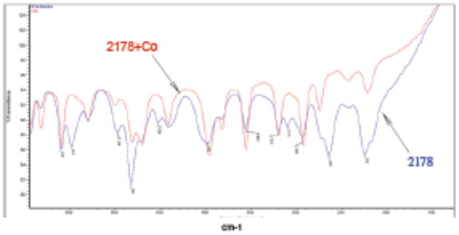

G)

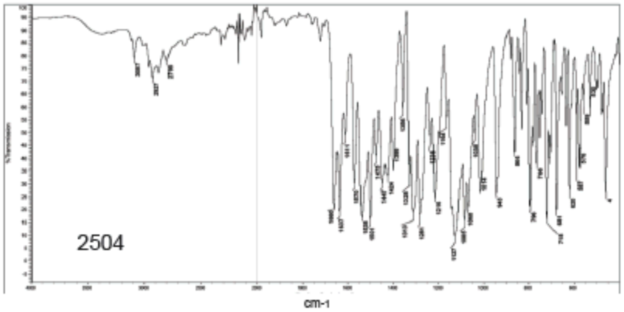

H)

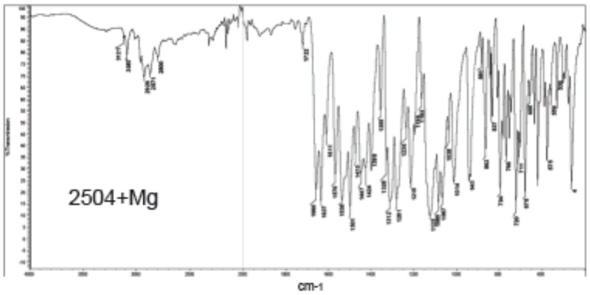

I)

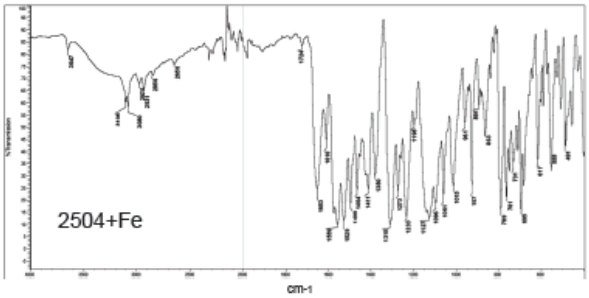

Fig S6. Infrared spectrum analysis of solid samples of compounds potentially combined with metals. IR spectra of (A) SAR1 alone, (B) SAR1 and magnesium, and (C) SAR1 and iron; (D) 2178 alone and (E) 2178 and magnesium; (F) far IR spectra of 2178 and cobalt (red) compared to 2178 alone (blue). IR spectra of (G) 2504 alone, (H) 2504 and magnesium, (I) 2504 and iron. Inset table summarizes relevant observations.
