# Supplementary material for: Bactericidal Disruption of Magnesium Metallostasis in Mycobacterium tuberculosis Is Counteracted by Mutations in the Metal Ion Transporter CorA
Source: mBio. 2019 Jul 9;10(4):e01405-19. doi: 10.1128/mBio.01405-19 (PMC6747715; doi:10.1128/mBio.01405-19)
Supplement: FIG S7 [file mBio.01405-19-sf007.pdf]

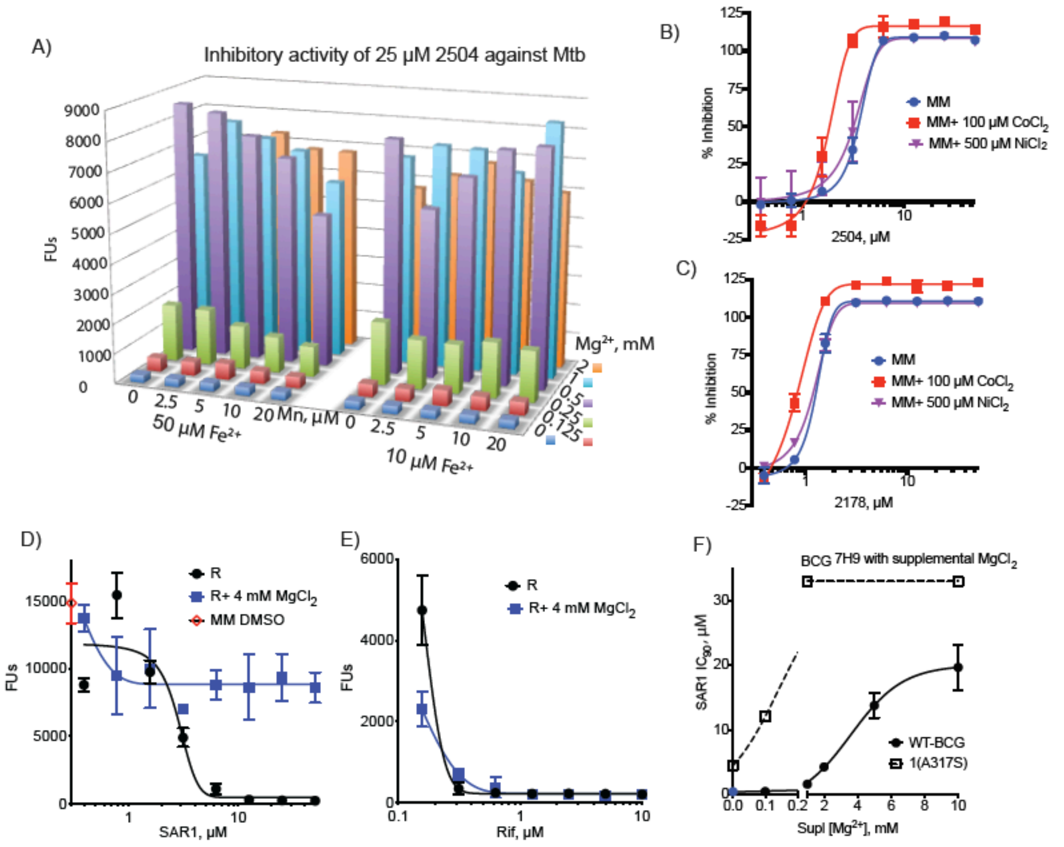

Fig. S7. Metal supplementation of growth media. (A) A checker board assay was executed to test the effect of varying metal concentrations on the inhibitory activity of 2504 at 25 µM on replicating Mtb. Complete minimal medium was supplemented with additional Mg^{2+} and Mn^{2+} at the indicated concentrations and at a final Fe^{2+} concentration of 50 µM or 10 µM. The single metal supplementation effect of 100 µM of $CoCl_2$ or 500 µM of $NiCl_2$ on the activity of (B) 2504 or (C) 2178. (D) SAR1 activity in response to excess magnesium in complete minimal medium as compared to (E) rifampicin (Rif); (F) effect of increasing concentrations of Mg^{2+} on the IC_{90} of BCG cells in response to SAR1 exposure. Data are means ± SE from one experiment representative of two independent experiments.
