# Supplementary material for: Bactericidal Disruption of Magnesium Metallostasis in Mycobacterium tuberculosis Is Counteracted by Mutations in the Metal Ion Transporter CorA
Source: mBio. 2019 Jul 9;10(4):e01405-19. doi: 10.1128/mBio.01405-19 (PMC6747715; doi:10.1128/mBio.01405-19)
Supplement: TEXT S1 [file mBio.01405-19-s0001.pdf]

Supplemental Methods

Bacterial growth conditions. Wild-type Mtb strain H37Rv (ATCC 25618) was grown in replicating conditions consisting of Middlebrook 7H9 broth supplemented with 10% oleic acid, dextrose and catalase (OADC) (BD Difco, USA), 0.5% glycerol, and 0.2% tyloxapol (Sigma) and incubated in 5% CO_2, 20% O_2 at 37 °C. *BCG* Pasteur IP1173P2 was cultivated in Middlebrook 7H9 medium (4.7 g/L Middlebrook 7H9 Broth, Difco) and supplemented with 0.4% (v/v) glycerol, 0.05% (v/v) Tween 80 and 10% (v/v) Middlebrook OADC enrichment, (0. 6% v/v oleic acid, 2 mg/mL dextrose, 5 mg/mL bovine serum albumin, 3 µg/mL catalase), or on solid medium 7H10 (19 g/L Middlebrook 7H10 Agar, Difco) adjusted to pH 6.6 and supplemented with 10% Middlebrook OADC enrichment and 0.5% glycerol. For non-replicating conditions, replicating Mtb cells were diluted in non-replicating medium composed of modified Sauton's base at a pH 5.0, butyrate as the carbon source, and 0.5 mM $NaNO_2$. The cells were then incubated in 5% CO_2 and 1.0% O_2 at 37 °C. In the nutrient starvation (Loebel) model of non-replication (Loebel, R. O., Shorr, E. & Richardson, H. B. *J Bacteriol* **26**, 167-200,1933), Mtb cells were incubated for 4 weeks in PBS-Tyl solution without $CaCl_2$ and $MgCl_2$ in 5% CO_2 and 20% O_2 at 37 °C ("starvation"). After a month of starvation, cells were exposed to compounds for 7 days, after which cell survival was assessed.

MIC and CARA assays. Minimum inhibitory concentration (MIC) values for Mtb cells were determined by serially diluting compounds in dimethylsulfoxide (DMSO) and dispensing into 96-well plate. Final DMSO concentrations did not exceed 1%. For the replicating MIC, bacterial suspensions were diluted to an OD_{580} of 0.01 and added to

the plate containing the compound. For non-replicating cells, NR MIC was determined as described (Warrier, T., et. al., *ACS Infect Dis* **1**, 580-585, 2015) by resuspending cells from the NR plates and transferred to a second plate containing supplemented 7H9 broth and incubated under replicating conditions for 10-12 days before recording the OD_{580}. For *BCG* MIC, 96-well plates were incubated for 6 days at 37 °C and 5% CO_2 in plastic bags. Alamar Blue (Promega) with 5% v/v Tween80 was added to the BCG cells to determine cell viability; fluorescence was measured (λex = 530 nm, λem = 590 nm) after 16-24 hrs. IC_{50} or IC_{90} were calculated using the in-house analysis program, Speedlogic (Sanofi). CARA plates were prepared and assayed as described (Gold, B., et. al., *Antimicrob Agents Chemother* **59**, 6521-6538, 2015; Gold, B., *J Vis Exp*.,118, 2016; doi: 10.3791/54690). Briefly, after 7 days of compound exposure, cells were resuspended and CARA plates containing agar with 4g/L charcoal were inoculated with 10 µL from the culture plate. After incubating the plates for 7 days (replicating assay) or 12-14 days (NR assay), 50 µl of resazurin solution was added to each well.

Whole cell screens. We conducted two whole-cell screens independently. 673,640 chemical compounds from the Sanofi library were screened at 10 µM against *M. smegmatis* under replicating conditions. In addition, 90,000 compounds from the Sanofi collection were screened at 10 µM using *M. tuberculosis* strain $mc^2$6220 (Δ*panCD*Δ*lysA*) (Larsen, M. H., et. al., *Vaccine* **27**, 4709-4717, 2009) under both replicating conditions and the non-replicating conditions of the 4-stress model (Gold, B., et. al., *Antimicrob Agents Chemother* **59**, 6521-6538, 2015).

Generation of resistant mutants. Resistant Mtb mutants for 2178, 2504, and 2150 were generated using 7H11 agar plates supplemented with 10% OADC and 0.5%

glycerol and made to contain 2X, 4X, 8X, or 10X the replicating MIC of the compound. These plates were inoculated with 10^9, 10^8, 10^7, or 10^6 CFUs. After 2 months of incubation, colonies were detected only on the 2X and 4X plates. SAR1 resistant *BCG* was selected on 7H10 at 2.5X and 10X the MIC. Resistant colonies were confirmed on 10X the SAR1 MIC. The genomes of seven independently isolated Mtb mutants were purified using the cetyltrimethylammonium bromide (CTAB, Sigma-Aldrich) extraction method (Amaro, A., et. al, *Lett Appl Microbiol* **47**, 8-11, 2008) followed by three phenol chloroform extractions to yield clean high quality DNA for whole genome sequencing. The three BCG genomes were isolated using Invitrogen Genomic DNA mini kit (Invitrogen, USA).

MD simulations. MD simulations of the wild type and the mutated A317S homopentamer were prepared, executed and analysed with Amber18 and AmberTools18 software packages (D.A. Case, I.Y. et. al., AMBER2018, University of California, San Francisco, 2018). CHARMM-GUI membrane builder (Lee, el. al., *J Chem Theory Comput* **12**, 405-413, 2016) was used for the embedding of the transmembrane part of CorA in the 1,2-dimyristoyl-sn-glycero-3-phosphocholine (DMPC) bilayer. Both systems were optimized with series of subsequent energy minimizations followed by low temperature short simulations, which gradually increased up to 300K simulations. The simulations were performed with the ff14SB and Lipid14 force fields (Dickson, C.J., et. al., *J Chem Theory Comput* **10**, 865-879, 2014; Maier, J. A., et. al., *J Chem Theory Comput* **11**, 3696-3713, 2015) . Explicitly solvated TIP3P waters (Jorgensen, W. L., Chandrasekhar, J., Madura, JD., *The Journal of Chemical Physics* **79**, 926, 1983) in NPT ensemble, and 2fs integration steps were used for

generation of more than 400 ns production runs in total with snapshots saved every 20 ps. Simulations were carried out with all 10 magnesium ions bound to their known sites in the intracellular part of the protein, and additional sodium ions were added for the system total charge neutralization.

ICP-MS. Agilent MassHunter for ICP-MS software was used for data reduction and processing. Instrument parameters are listed below. An independent reference material was analyzed immediately after calibration to verify instrument accuracy. All QA/QC samples met a priori acceptance criteria. All samples were diluted fifteen-fold in 2% (v/v) nitric acid to normalize acid content with the calibration standards. Final graphical values were expressed as concentrations in the 30% nitric acid extract in which ppm (μg/ mL) or ppb (ng/ mL) were converted to μM.

Agilent 7800 ICP-MS Instrument Settings

	Hydrogen Mode	Helium Mode
RF Power (Watts)	1600	1600
Sample Depth (mm)	8	8
Nebulizer Gas (L\min)	0.75	0.75
Dilution Gas (L\min)	0.4	0.4
OctP Bias (V)	-20	-20
KED (V)	4	4
Cell Gas Flow (ml\min)	6	5

Metal complexation using Infrared spectroscopy. We attempted to isolate potential metal complexes by adding a solution of 2178 or 2504 (50 mg, 1eq) in 20 mL dichloromethane (DCM) and 3 mL of methanol to a solution of magnesium chloride hexahydrate (27.5 mg, 1 eq) or cobalt (II) chloride hexahydrate (31.5 mg, 1 eq) in 1 mL of methanol. The resulting solution was left open in a fume hood for 4-6 hours until a

suspension formed after about half the volume evaporated. The solid was filtered and vacuum dried. Characterization was performed using Infrared (IR) spectrometry and elemental analysis (EA). Compound and compound-metal complex spectra were generated in a Nexus FT-IR spectrometer (Thermo Fisher) using solid state samples prepared from cesium iodide chips for the far IR spectra or by Attenuated Total Reflection (ATR) for the medium IR spectra. Elemental analysis was carried out by introducing 1 mg of sample into a reaction tube maintained at 1000 °C using a Flash 2000 system (Thermo Fisher). Combustion gases (CO_2, H_2O, N_2O and SO_2) were then separated and dosed by chromatography. Water content was determined by Karl-Fischer coulometric titration.
